# Evaluating the Safety and Efficacy of Malaria Preventive Measures in Pregnant Women with a Focus on HIV Status: A Systematic Review and Network Meta-Analysis

**DOI:** 10.3390/jcm14103396

**Published:** 2025-05-13

**Authors:** Muayad Albadrani, Heba M. Eltahir, Ahmad Bakur Mahmoud, Mekky M. Abouzied

**Affiliations:** 1 Department of Family and Community Medicine and Medical Education, College of Medicine, Taibah University, Madinah 42353, Saudi Arabia; 2 Health and Life Research Center, Taibah University, Madinah 42353, Saudi Arabia; 3 Department of Pharmacology and Toxicology, College of Pharmacy, Taibah University, Madinah 42353, Saudi Arabia; 4 Department of Clinical Laboratory Sciences, College of Applied Medical Sciences, Taibah University, Madinah 42353, Saudi Arabia; 5 Department of Biochemistry, Faculty of Pharmacy, Minia University, Minia 61519, Egypt

**Keywords:** preventive measures, pregnancy, HIV, malaria, network meta-analysis

## Abstract

**Background and Objectives:** Malaria poses significant threats to pregnant women, particularly in endemic regions. Preventive measures against it include insecticide-treated bed nets, intermittent preventive treatment, and various supplements. We aimed to assess and compare the safety and effectiveness of malaria preventive measures in pregnant women, considering their HIV status. **Methods:** We conducted a systematic search of PubMed, the Cochrane Library, Scopus, Embase, and Web of Science through January 2024. A network meta-analysis was performed using R 4.3.3 software on 35 studies (50,103 participants). **Results:** In HIV-positive pregnant women, Co-trimoxazole with dihydroartemisinin significantly reduced malaria incidence compared to Co-trimoxazole alone (RR = 0.45, 95% CI [0.30; 0.68]) and sulfadoxine–pyrimethamine (SP) (RR = 0.14, 95% CI [0.04; 0.48]). Mefloquine was also effective compared to controls and SP. In HIV-negative women, azithromycin–piperaquine significantly reduced infections compared to SP, bed nets, and controls (RR = 0.03, 95% CI [0.00; 0.83]; RR = 0.03, 95% CI [0.00; 0.86]; and RR = 0.03, 95% CI [0.00; 0.77], respectively). **Conclusion:** Different combinations of preventive measures show varying effectiveness based on HIV status. Co-trimoxazole with dihydroartemisinin and mefloquine are effective for HIV-infected pregnant women, while azithromycin–piperaquine and mefloquine work well for those without HIV. Customized prevention strategies considering HIV status are crucial for optimal protection.

## 1. Introduction

Malaria is a significant contributor to illness and death on a global scale, particularly impacting children below the age of five and pregnant women, who are the most vulnerable populations [1]. In 2022, the global number of malaria cases was estimated to be 249 million, surpassing the pre-pandemic figure of 233 million cases recorded in 2019 by an additional 16 million. Besides the challenges posed by the COVID-19 pandemic, the worldwide efforts to address malaria have encountered various emerging threats, including drug and insecticide resistance, humanitarian crises, limitations in resources, the impacts of climate change, and delays in implementing programs, especially in nations heavily burdened by the disease [1,2,3]. Malaria places a significant health and socioeconomic burden on global populations, with approximately 3.2 billion individuals facing the risk of malaria infection [4]. From 2000 to 2015, there was a 37% decline in global malaria incidence, progress attributed to economic development and urbanization in numerous endemic nations [4,5]. Additionally, there was a notable rise in investments aimed at addressing malaria, resulting in increased preventive measures, enhanced diagnostics, and improved treatment strategies [6].

Vector control is crucial in the efforts to control and eliminate malaria. The ability of vectors to transmit parasites and their susceptibility to control measures vary among mosquito species and are influenced by local environmental factors. Current prevention practices predominantly rely on personal preventive measures, which aim to minimize contact between adult mosquitoes and humans. Notably, these measures include two types of insecticide-treated nets (ITNs): long-lasting insecticidal nets (LLINs) with insecticide embedded during manufacturing for prolonged effectiveness, and regular ITNs requiring insecticide reapplication every six months. Another approach is indoor residual spraying (IRS), involving the application of insecticides on household walls [7].

Furthermore, anti-malarial chemoprophylaxis is employed for malaria prevention in children and pregnant women. Sulfadoxine–pyrimethamine (SP), mefloquine (MQ), amodiaquine (AQ), dihydroartemisinin–piperaquine (DP), and artesunate (AS) are commonly used prophylactic drugs, offering the advantage of achieving full prophylactic effects with a single dose [8,9]. Several less commonly employed measures in malaria prevention include insecticide-treated curtains (ITCs), mosquito coils, insecticide-treated hammocks, and insecticide-treated tarpaulins. Despite a global decrease in malaria incidence, the most effective common preventive interventions for malaria infection remain unclear. Identifying the most effective interventions is essential for prioritizing resources. A single comparative study evaluating preventive efficacy across insecticide-treated nets (ITNs), indoor residual spraying (IRS), and prophylactic drugs (PDs) found that IRS is as effective as ITNs in reducing malaria-attributable mortality in children [9]. While the WHO previously endorsed sulfadoxine–pyrimethamine (SP), the diminishing effectiveness of SP in addressing symptomatic malaria over the years has raised apprehensions regarding its appropriateness for extended use in intermittent preventive treatment.

Malaria during pregnancy is a significant global health problem, particularly in areas with moderate-to-high transmission. Pregnant women are more susceptible to malaria due to reduced immunity, and HIV co-infection further increases their vulnerability. The WHO recommends a package of interventions for preventing and controlling malaria during pregnancy. For pregnant women in areas with moderate-to-high transmission of Plasmodium falciparum, the WHO recommends intermittent preventive treatment with SP, starting in the second trimester. For HIV-positive pregnant women, daily Co-trimoxazole (CTX) prophylaxis is the standard. These recommendations are crucial for tailoring malaria preventive strategies to the specific needs of pregnant women, considering their HIV status. Preventive measures include ITNs, intermittent preventive treatment, and various supplements.

We aim to evaluate and compare the safety and efficacy of various preventive strategies employed to combat malaria in pregnant women, with a specific consideration of their HIV status. This assessment encompasses an exploration of different interventions, such as insecticide-treated nets (ITNs), indoor residual spraying (IRS), and anti-malarial chemoprophylaxis, in order to discern their comparative advantages and potential drawbacks in mitigating the risk of malaria infection during pregnancy. Additionally, we seek to examine how HIV status influences the effectiveness and safety profiles of these preventive measures, aiming to provide nuanced insights into the optimal strategies for malaria prevention in pregnant women living with HIV.

## 2. Methods

We conducted our systematic review and network meta-analysis in adherence with the preferred reporting items for systematic reviews and meta-analyses (PRISMA) guidelines for network meta-analysis. Also, we followed the guidelines outlined in the Cochrane Handbook for systematic reviews throughout this study [10,11].

### 2.1. Searching Databases and Keywords

We searched four databases in January 2024 (PubMed, Cochrane Library, Web of Science, and Scopus), and two individual authors searched. The detailed search string used for each database is provided in Supplementary Table S1. We carried out our search without imposing limitations on time or language and supplemented it by manually examining the references of the studies included in our analysis.

### 2.2. Eligibility Criteria and Study Selection

We included all eligible studies that used preventive measures against malaria in pregnant women with or without HIV. We included all possible interventions, and we compared the results of each intervention regarding maternal and neonatal outcomes, in addition to safety outcomes such as abdominal pain, dizziness, headache, nausea, vomiting, neonatal deaths, preterm birth, and stillbirth. We excluded cohorts, letters, abstracts that did not provide information, case controls, and case series. Titles and abstracts were initially screened, followed by a thorough examination of the full texts of potentially relevant studies to assess eligibility and determine the final set of included studies.

### 2.3. Data Extraction

We extracted the following data from the included studies. (A) Baseline data: study ID, follow-up duration site, study design, maternal age, gestational age, gravid. (B) Summary data, including arm description, diagnostic tools, primary endpoint, and conclusion. (C) The outcomes that we included in our analysis were as follows: 1—incidence of malarial infection; 2—maternal anemia at delivery; 3—low birth weight (less than 2.5 kg); 4—abdominal pain incidence; 5—headache; 6—nausea; 7—vomiting; 8—neonatal deaths; 9—preterm birth; and 10—stillbirth. Two reviewers independently extracted the data to ensure accuracy and consistency. Any discrepancies between the reviewers were resolved through consultation with a third reviewer. This approach ensured that the data extraction process was thorough and reliable, contributing to the robustness of our findings.

### 2.4. Quality Assessment

We employed the Cochrane risk of bias tool [12] to evaluate the quality of the included RCT studies, assessing various domains such as the random sequence generation, concealed allocation, blinding of participants and personnel, blinding of outcome assessors, handling of incomplete data, selective reporting, and other relevant aspects. Each domain was independently evaluated by two authors, and conflicts were resolved through consultation with a third author. We also used quasi-experimental study design risk of bias assessment to assess two studies [13].

### 2.5. Statistical Analysis

We utilized the netmeta package in R 4.3.3 software to perform a frequentist network meta-analysis. Network plots were created to visually display the interventions and their direct and indirect comparisons, helping to understand the structure and strength of the network. The assumptions of NMA, including transitivity, consistency, and similarity, were carefully considered. Transitivity was assumed based on the similarity of patient characteristics, interventions, and outcomes across studies, allowing for valid indirect comparisons. Consistency was assessed using node-splitting methods to compare direct and indirect evidence within the network, ensuring the robustness of our findings. Similarity was ensured by including studies that were comparable in design and execution. The reference treatment was selected based on its common use and relevance in the included studies, providing a stable and consistent comparator across the network. A random-effects model was used to account for variability among studies and ensure a comprehensive analysis. Outcomes were pooled using both direct and indirect evidence to provide a comprehensive estimate of the relative effects of the interventions. Heterogeneity among studies was assessed using the Chi-squared test (Q2) and I-squared test, with significant heterogeneity defined as I2 > 50% or a *p*-value < 0.1. A random-effects model was applied to address significant heterogeneity. P-scores were calculated to rank the interventions based on their effectiveness and safety profiles, providing a quantitative measure of the relative performance of each intervention. By incorporating these elements, we aimed to provide a robust and transparent analysis of the effectiveness and safety of malaria preventive measures in pregnant women.

## 3. Results

### 3.1. Literature Search

**Figure 1:**
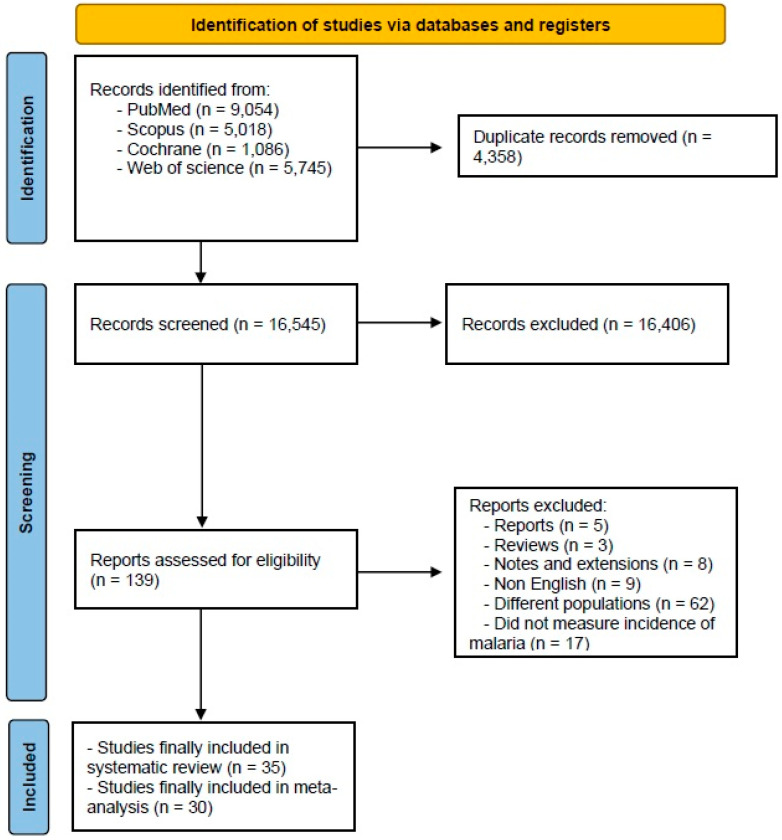
A flowchart depicting the selection process of the studies included in the meta-analysis. The diagram follows the PRISMA guidelines, illustrating the number of records identified, screened, excluded, and included in the final analysis.

The initial database search yielded 20,903 records, reduced to 16,545 after removing 4358 duplicates. Subsequent title and abstract screening identified 139 studies for full-text assessment, ultimately including 35 studies [14,15,16,17,18,19,20,21,22,23,24,25,26,27,28,29,30,31,32,33,34,35,36,37,38,39,40,41,42,43,44,45,46,47,48] in the systematic review; 30 of these studies of were included in the analysis (Figure 1, PRISMA).

### 3.2. Summary and Baseline Characteristics of Included Studies

Our network meta-analysis comprised nine studies that were about pregnant women with HIV, while the remaining included studies were about HIV-negative pregnant women. The studies included a total of 50,103 participants, representing a diverse population from multiple countries, with a predominant focus on the African region. The countries included were Mali, Pakistan, Australia, Kenya, Thailand, the Republic of Congo, Gambia, Benin, Burkina Faso, Ghana, Nigeria, Tanzania, Mozambique, Uganda, and Malawi. Most participants were within the maternal and gestational age range of 20 to 30 years. The follow-up duration across the studies varied from three months to two years. Various diagnostic tools were employed: quantitative PCR, nested PCR, loop-mediated isothermal amplification, thick and thin blood smears stained with Giemsa stain, microscopic examination, and targeted next-generation sequencing for molecular markers. The primary endpoint in most studies was the incidence of malarial infections. The studies included in our analysis covered a period from 1993 to 2024 (Table 1 and Table 2).

### 3.3. Risk of Bias

**Figure 2:**
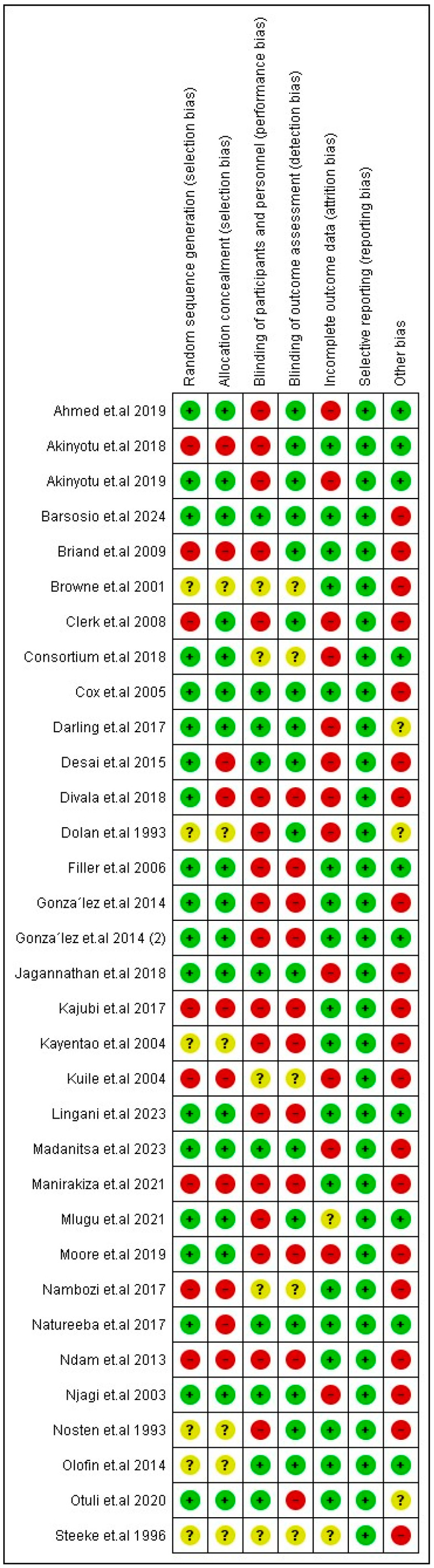
Risk of bias of included RCTs. Symbols: green “+” = positive association; red “−” = negative association; yellow “?” = unclear or insufficient data.

Most of the RCTs were low-risk regarding randomization and allocation processes. Moreover, all the RCTs were low-risk regarding reporting bias. However, only two of the included studies were low-risk regarding all the aspects of risk of bias except for other bias [17,42]. They were both low-risk for all aspects of risk of bias except for one aspect, while most of the other included studies were at a high risk of bias. Analysis of the two other quasi-experimental studies showed that Kumar et al., 2020 [27], was fair in quality and Roh et al., 2022 [39], was good in quality. All the details about the risk of bias and quality assessment are presented in Figure 2 and Supplementary Table S2, respectively.

### 3.4. Outcomes

(A) Preventive measures for pregnant women with HIV

1. Incidence of malarial infection.

**Figure 3:**
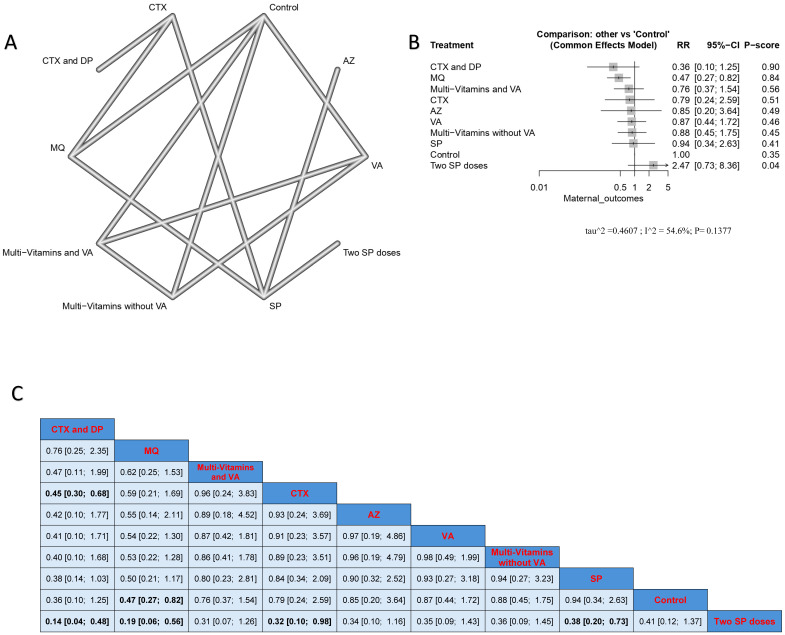
Incidence of malarial infection in pregnant women with HIV. (**A**) Network graph showing direct evidence between evaluated interventions. (**B**) Forest plot comparing all interventions. (**C**) League table representing network meta-analysis estimates for all interventions’ comparisons.

The combination of CTX with DP caused a significantly lower incidence rate when compared to CTX alone (RR = 0.45, 95% CI [0.30; 0.68]) and SP doses (RR = 0.14, 95% CI [0.04; 0.48]). The MQ intervention had a significantly lower incidence of malarial infection when compared with controls and two SP doses, and the results were (RR = 0.47, 95% CI [0.27; 0.82]), and (RR = 0.19, 95% CI [0.27; 0.82]), respectively. Also, CTX had a significantly lower incidence rate than the two SP doses. Nevertheless, the results were heterogeneous. The best three treatments, according to p-score, were the combination of CTX with DP, MQ, and the combination of multivitamins and VA (Figure 3).

2. Maternal anemia at delivery.

No significant difference was found among interventions regarding maternal anemia at delivery. According to the p-scores, the interventions that induced anemia the least were arranged as two SP doses, followed by SP, CTX, and MQ (Supplementary Figure S1).

3. Low birth weight.

We found no significant difference between the different interventions regarding low birth weight. The lowest incidence of a low birth weight of less than 2.5 kg, according to p-score, was observed with AZ, followed by MQ, followed by two SP doses (Supplementary Figure S2).

### 3.5. Safety Outcomes in Pregnant Women with HIV and Preventive Measures 

No significant difference was detected among the different interventions regarding dizziness and headache. A significantly lower incidence of vomiting was seen in CTX treatment compared to MQ (RR = 0.07, 95% CI [0.01; 0.30]) (Supplementary Figures S3–S5, respectively).

Regarding preterm birth and stillbirth, there was no significance among the interventions. According to the p-scores, SP was associated with the lowest incidence of preterm birth, while MQ was associated with the lowest stillbirth incidence (Supplementary Figures S6 and S7, respectively).

(B) Pregnant women taking preventive measures without having HIV.

1. Incidence of malarial infection.

**Figure 4:**
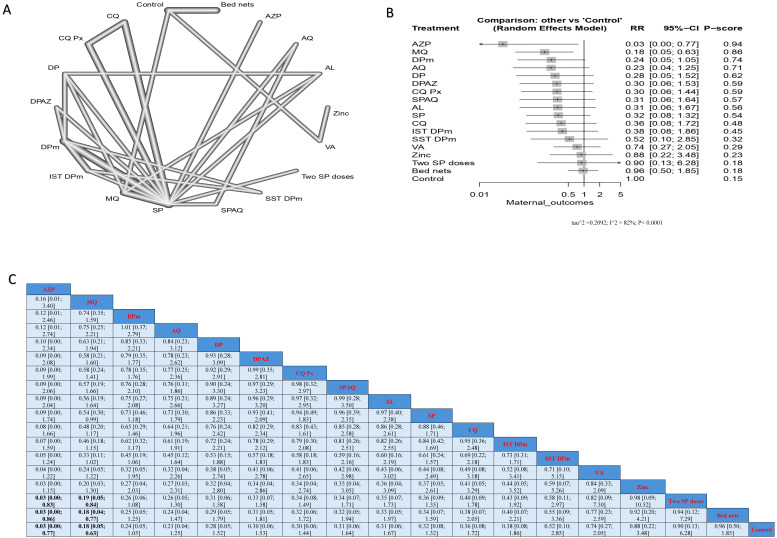
Incidence of malarial infection in pregnant women without HIV. (**A**) Network graph showing direct evidence between evaluated interventions. (**B**) Forest plot comparing all interventions. (**C**) League table representing network meta-analysis estimates for all interventions’ comparisons.

AZP significantly reduced infection when compared with two SP doses, bed nets, and controls; the results were (RR = 0.03, 95% CI [0.00; 0.83]), (RR = 0.03, 95% CI [0.00; 0.86]), and (RR = 0.03, 95% CI [0.00; 0.77]), respectively. Similarly, MQ significantly reduced infection when compared with two SP doses, bed nets, and controls; the results were (RR = 0.19, CI = 95% CI [0.05; 0.84]), (RR = 0.18, 95% CI [0.04; 0.77]), and (RR = 0.18, 95% CI [0.05; 0.63]), respectively. Nevertheless, the top treatments, according to p-score, that reduced malarial infection in patients without HIV were AZP, MQ, and DPm, followed by AQ. On the other hand, the least effective interventions were bed nets, two SP doses, and zinc (Figure 4).

2. Maternal anemia at delivery.

Comparing the different interventions regarding maternal anemia at delivery, only MQ showed a significant decrease in the incidence compared to CQ (RR = 0.54, 95% CI [0.31; 0.94]) (Supplementary Figure S8).

3. Low birth weight.

No significant difference was detected among the different interventions regarding neonatal birth weight. However, the lowest incidence of a neonatal birth weight of less than 2.5 kg, according to p-score, was found in IST DPm, followed by SST DPm and MQ (Supplementary Figure S9).

### 3.6. Safety Outcomes in Pregnant Women Taking Preventive Measures Without Having HIV

**Figure 5:**
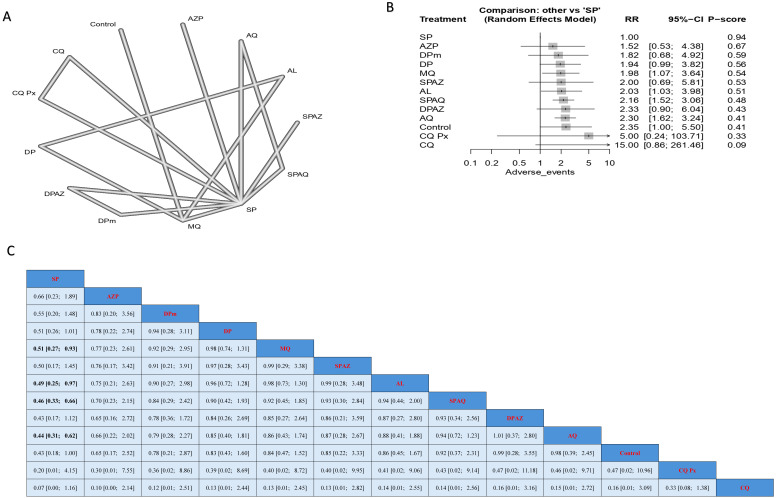
Incidence of abdominal pain in pregnant women without HIV. (**A**) Network graph showing direct evidence between evaluated interventions. (**B**) Forest plot comparing all interventions. (**C**) League table representing network meta-analysis estimates for all interventions’ comparisons.

Regarding the incidence of abdominal pain, SP had a significantly lower incidence when compared to MQ, SPAQ, and AQ; the results were (RR = 0.51, 95% CI [0.27; 0.93]), (RR = 0.46, 95% CI [0.33; 0.66]), and (RR = 0.44, 95% CI [0.31; 0.62]), respectively (Figure 5).

**Figure 6:**
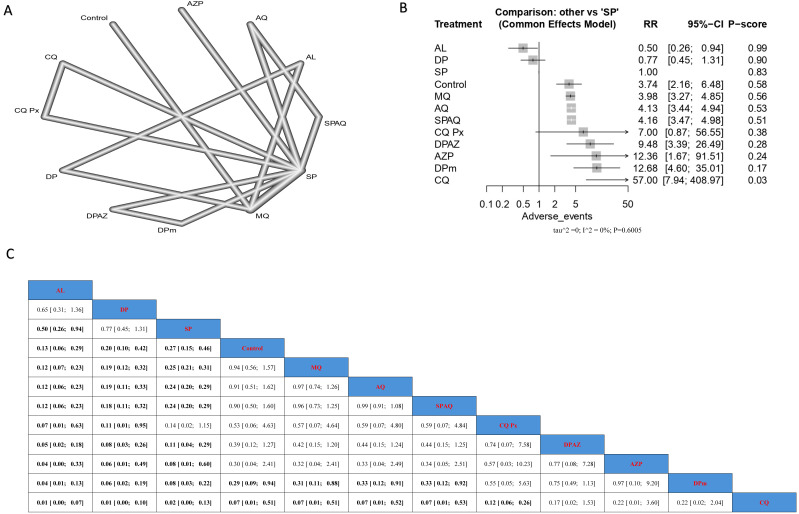
Incidence of dizziness in pregnant women without HIV. (**A**) Network graph showing direct evidence between evaluated interventions. (**B**) Forest plot comparing all interventions. (**C**) League table representing network meta-analysis estimates for all interventions’ comparisons.

Regarding the incidence of dizziness, AL was associated with the lowest incidence of dizziness and was significant compared to all arms except for DP. It should be noted that DP and SP were associated with lower incidences of dizziness than most other interventions (Figure 6). DPm had the highest incidence of nausea compared to other arms, where its effect was significantly different from SP (RR = 0.05), AQ (RR = 0.10), and AZP (RR = 0.11). Regarding vomiting, the intervention of SP was associated with the lowest incidence of vomiting, with a significant result compared to MQ and AQ, with (RR = 0.23) and (RR = 0.28), respectively. No significant difference could be detected among the interventions regarding headache incidence rate as a side effect (Supplementary Figures S10–S12, respectively).

### 3.7. Neonatal Deaths

**Figure 7:**
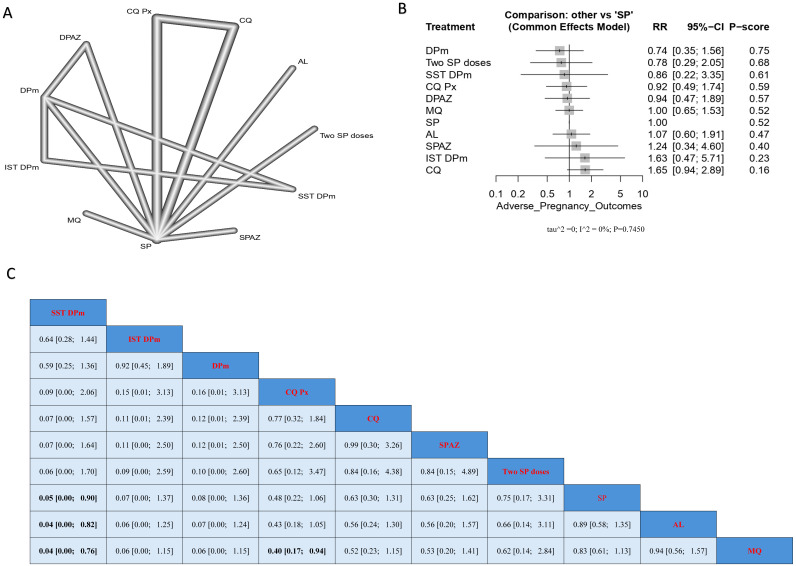
Incidence of neonatal deaths in pregnant women without HIV. (**A**) Network graph showing direct evidence between evaluated interventions. (**B**) Forest plot comparing all interventions. (**C**) League table representing network meta-analysis estimates for all interventions’ comparisons.

Treatment with CQ resulted in the highest incidence of neonatal death. SST DPm showed a significantly lower incidence of neonatal death when compared with SP, AL, and MQ, and the results were (RR = 0.05, 95% CI [0.00; 0.90]), (RR = 0.04, 95% CI [0.00; 0.82]), and (RR = 0.04, 95% CI [0.00; 0.76]), respectively. It should be noted that the lowest incidence of neonatal deaths based on the p-score was observed with DPm treatment, followed by two SP doses, and then SST DPm (Figure 7).

### 3.8. Stillbirth

The highest incidence of stillbirth was observed in the case of MQ treatment. In contrast, both SST DPm and CQ Px showed significantly lower stillbirth incidence when compared with MQ, and the results were (RR = 0.04, 95% CI [0.00; 0.76]) and (RR = 0.4, 95% CI [0.17; 0.94]), respectively. The lowest incidence of stillbirth, according to p-score, was observed with SST DPm, followed by IST DPm and then DPm (Supplementary Figure S13).

## 4. Discussion

Travelers visiting high-risk malaria areas, particularly pregnant women with or without HIV, should consider taking anti-malarial medication. However, chemoprophylaxis is not advisable for destinations with sporadic malaria cases and a low transmission risk. The choice of medication depends on factors such as local drug resistance, travel duration, medical history, allergies, and potential side effects. Additionally, individuals can reduce infection risk by taking preventive measures, including limiting outdoor activities, using insect repellents, and using insecticide-treated bed nets. Our study emphasizes the effectiveness of various preventive measures against malaria in both HIV-positive and -negative individuals. Combinations like Co-trimoxazole with dihydroartemisinin and mefloquine demonstrate efficacy in reducing malaria incidence compared to other interventions. Meanwhile, azithromycin with piperaquine and dihydroartemisinin is effective in HIV-negative individuals. However, safety concerns exist for interventions like mefloquine in pregnant women. Multivitamin supplementation and azithromycin also hold promise. Overall, tailored preventive strategies considering factors like HIV status and pregnancy are crucial.

Before the widespread implementation of antiretroviral therapy, Co-trimoxazole (CTX) was a cost-effective, broad-spectrum antimicrobial medication extensively utilized in developing nations. It played a crucial role in decreasing morbidity and mortality among individuals, including both adults and children, living with HIV by preventing various infections such as bacterial infections, diarrhea, malaria, and Pneumocystis pneumonia, even in the face of prevalent microbial resistance [49]. According to previous studies, CTX prophylaxis significantly reduces early mortality rates [49,50,51]. Since 2001, the World Health Organization (WHO) has endorsed artemisinin-based combination therapies (ACTs) as the primary treatment for uncomplicated *P. falciparum* malaria [7]. Artemisinin and its derivatives are well known for their strong anti-malarial properties and have been widely adopted for clinical use in regions where malaria is endemic. In laboratory settings, the artemisinin concentration required to inhibit 50% of Plasmodium falciparum growth ranges from 3 to 30 μg/L [51]. The combination of CTX and artemisinin-based combination therapies for prevention and treatment has shown effectiveness against malaria in HIV-positive patients. CTX reduces morbidity and mortality in individuals with HIV by preventing various infections, including malaria. Meanwhile, ACTs, endorsed by the World Health Organization since 2001, are potent in treating uncomplicated *P. falciparum* malaria. Combining these drugs offers a synergistic approach, enhancing malaria management strategies, especially in endemic regions [42,52].

Mefloquine is widely recognized for its high efficacy in preventing and treating malaria. It is considered one of the most effective anti-malarial drugs available, particularly in regions where malaria parasites have not developed resistance to it. When used correctly and combined with other preventive measures, mefloquine can provide robust protection against malaria infection [53,54]. The World Health Organization (WHO) permits the use of mefloquine for pregnant women during the second and third trimesters, while some authorities, such as the Centers for Disease Control and Prevention (CDC), extend this approval to the first trimester [55]. In the event of accidental pregnancy while using mefloquine, termination is not recommended. Additionally, mefloquine chemoprophylaxis is considered safe during breastfeeding. Studies indicate that mefloquine is a viable option for other high-risk groups, including long-term travelers, visiting friends and relatives (VFR) travelers, and families with young children. Despite negative media portrayal, extensive pharmaco-epidemiological investigations have demonstrated that serious adverse events associated with mefloquine are rare [56]. In our study, we found that mefloquine is not highly ranked in terms of safety outcomes in pregnant women without HIV, and it may even increase the number of stillbirth infants significantly.

The use of multivitamin supplements containing vitamin B complexes, C, and E, has been observed to decelerate disease progression and lower the occurrence of HIV-associated complications such as dysentery and acute upper respiratory infections in HIV-positive women. However, it remains unclear whether multivitamins impact malaria susceptibility in HIV-positive women. Research conducted among children indicates that multivitamin supplementation may reduce the incidence of clinical malaria [46,57].

Azithromycin has been investigated as a potential anti-malarial agent due to its slow yet potent activity against malaria parasites, targeting the apicoplast organelle [58,59]. It is considered the most potent anti-malarial macrolide, demonstrating significant activity against cultured Plasmodium falciparum after extended in vitro exposure [59]. In treating uncomplicated falciparum malaria, combinations such as artesunate plus azithromycin have shown improved efficacy compared to artesunate alone. However, they are less effective than combinations including mefloquine or dihydroartemisinin [60]. Studies assessing azithromycin in combination with chloroquine have produced mixed results, with some showing promising efficacy while others find it inferior to alternative treatments like artemether–lumefantrine [60,61].

Our study possesses several strengths, notably in comprising most of our included studies, which were randomized controlled trials and considered the gold standard in evidence quality. Our study marks the first network meta-analysis to systematically compare various outcomes between pregnant women with HIV and those without HIV. With a substantial participant pool of 50,103 individuals across 35 studies, our study provides comprehensive insights into the efficacy and safety of preventive measures against malaria in pregnancy. Our findings promise to inform future decision-making regarding selecting appropriate preventive strategies for malaria infection. However, it is essential to acknowledge certain limitations. A prevalent risk of bias compromised the overall quality of the included studies. Furthermore, factors inherent to pregnancy may confound the association between preventive measures and malaria incidence. Adverse events observed during the study period may not solely be attributable to malaria infection or preventive measures but could also be influenced by the physiological changes associated with pregnancy. Additionally, variations in malaria detection techniques may introduce heterogeneity into our analysis.

## 5. Conclusions

Our study highlights the efficacy of various preventive measures against malaria in both HIV-positive and -negative individuals. Combinations like Co-trimoxazole with dihydroartemisinin and mefloquine show effectiveness in reducing malaria incidence compared to other interventions, while azithromycin with piperaquine and dihydroartemisinin are effective in HIV-negative individuals compared to other interventions. However, concerns exist regarding the safety of certain interventions, such as mefloquine, in pregnant women. Multivitamin supplementation and azithromycin also show promise, but further research is needed to confirm their effectiveness. Overall, tailored preventive strategies considering factors like HIV status and pregnancy are essential, with future research focusing on optimizing interventions while ensuring patient safety.

## Figures and Tables

**Table 1:** Baseline characteristics of included studies.

Study ID	Study Arms, n (%)	Site	Study Design	Maternal Age, (Mean ± SD) Year	Gestational Age, (Mean ± SD) Weeks	Gravida, n (%)	Follow-Up Duration (Months)	Arm Description
Ahmed et al., 2019 [14]	SST DPm, 744 (32.65)	Indonesia	RCT (ISRCTN 34010937)	27 ± 6.2	23.9 ± 5	1. G1, 201 (27) 2. G2, 194 (26.1) 3. ≥G3, 349 (46.9)	Mean (3.1)	Single screening and treatment during pregnancy with dihydroartemisinin–piperaquine
	IST DPm, 854 (37.47)			26.7 ± 6.4	23.4 ± 4.8	1. G1, 264 (30.9) 2. G2, 284 (33.3) 3. ≥G3, 306 (35.8)		Intermittent screening and treatment during pregnancy with dihydroartemisinin–piperaquine
	DPm, 681 (29.88)			26.8 ± 6.1	23.9 ± 4.6	1. G1, 202 (29.7) 2. G2, 235 (34.5) 3. ≥G3, 244 (35.8)		Intermittent preventive treatment during pregnancy with dihydroartemisinin–piperaquine
Briand et al., 2009 [15]	MQ, 802 (50.01)	Benin	RCT (NCT00274235)	25 ± 5.4	24 ± 2.8	G1, 216 (27)	5.52 (SD 0.64)	SP (1500 mg of sulfadoxine and 75 mg of pyrimethamine per dose) plus daily ferrous (400 mg) and folic (5 mg) acid
	SP, 799 (49.99)			25 ± 5.4	24 ± 2.8	G1, 215 (27)	7.59 (SD 0.53)	MQ (15 mg/kg per dose) plus daily ferrous (400 mg) and folic (5 mg) acid
Clerk et al., 2008 [16]	SP, 693 (36.65)	Ghana	RCT (NCT00146783)	21.1 ± 3.5	24 ± 3.9	G1 or G2	At least 1	Single dose of SP (1500 mg sulfadoxine and 75 mg pyrimethamine)
	AQ, 503 (26.6)			21.6 ± 3.5	24 ± 4			Full treatment course of AQ (25 mg/kg) over 3 days
	SPAQ, 695 (36.75)			21.4 ± 3.5	23.9 ± 3.9			SPAQ given over 3 days
Cox et al., 2005 [17]	VA, 48 (48.98)	Ghana	RCT (not registered)	21 ± 2.9	17 ± 4.3	G1, 98 (100)	Up to 3.68	Capsules were given weekly containing 10,000 IU of vitamin A as retinyl palmitate in groundnut oil, plus tocopherol as preservative.
	Control, 50 (50.02)			21 ± 2.9	15 ± 5.6			Groundnut oil and tocopherol only in placebo capsules
Darling et al., 2017 [18]	VA, 697 (27.79)	Tanzania	RCT (NCT0111478)	23 ± 5	10 ± 2.4	G1, 321 (46)	Up to 10	2500 IU vitamin A
	VA and Zinc, 707 (28.19)			22.7 ± 3.7	10 ± 2.3	G1, 346 (49)		Both 2500 IU vitamin A and 25 mg zinc
	Zinc, 694 (27.67)			23 ± 4.8	10.1 ± 2.4	G1, 333 (48)		25 mg zinc (as zinc sulfate)
	No Zinc, 710 (28.31)			22 ± 7.4	10 ± 2.3	G1, 333 (47)		Placebo
Desai et al., 2015 [19]	IST DPm, 515 (33.34)	Kenya	RCT (NCT01669941)	23.4 ± 5.9	22.9 ± 4.7	1. G1/G2, 267 (51.8) 2. G3+, 248 (48.2)	Up to 9	Standard 3-day course of DP (2, 3, or 4 tablets/day for 24–35.9, 36–74.9, and ≥75 kg)
	DPm, 514 (33.33)			23.4 ± 5.5	23 ± 4	1. G1/G2, 263 (51.2) 2. G3+, 251 (48.8)		Standard 3-day course of DP (40 mg/320 mg/tablet)
	SP, 514 (33.33)			23.5 ± 6	22.8 ± 4.4	1. G1/G2, 292 (56.8) 2. G3+, 222 (43.2)		Three tablets of quality-assured SP (500/25 mg/tablet)
Divala et al., 2018 [20]	CQ Px, 300 (33.33)	Malawi	RCT (NCT01443130)	20.4 ± 3.6	22.5 ± 2.2	G1 or G2	At least 6	600 mg of chloroquine at enrolment followed by 300 mg once every week up to delivery
	CQ, 300 (33.33)			20.7 ± 3.2	22.2 ± 2.2			Two treatments of chloroquine given as 600 mg on day 1, 600 mg on day 2, and 300 mg on day 3 at least four weeks apart during pregnancy
	SP, 300 (33.34)			20.4 ± 3.1	22 ± 2.1			1500 mg sulfadoxine and 75 mg pyrimethamine twice at least four weeks apart during pregnancy
Dolan et al., 1993 [21]	Bed nets, 223 (65.4)	Thailand	RCT (not registered)	26 ± 6.5	NR	G1, 40 (17.94)	Up to 12	Permethrin-impregnated bed net (PIB) or untreated bed net (NIB)
	Control, 118 (34.6)			25.49 ± 6.04		G1, 28 (23.73)		No study bed net or family-sized non impregnated bed net
Filler et al., 2006 (Non-HIV) [22]	SP, 216 (50)	Malawi	RCT (NCT00126906)	19.5 ± 2.6	22.3 ± 3.6	G1, 131 (60.6)	At least 6	Two-dose SP, with directly observed treatment doses (1500 mg sulfadoxine and 75 mg pyrimethamine)
	Two SP doses, 216 (50)			19.9 ± 2.4	21.9 ± 3.7	G1, 115 (53.2)		Monthly SP, with directly observed treatment doses at enrollment and then monthly until delivery
Filler et al., 2006 (HIV) [22]	SP, 135 (50.75)	Malawi	RCT (NCT00126906)	21.6 ± 2.7	21.9 ± 3.8	G1, 59 (43.7)	At least 6	Two-dose SP, with directly observed treatment doses (1500 mg sulfadoxine and 75 mg pyrimethamine)
	Two SP doses, 131 (49.25)			21.6 ± 3.8	22.0 ± 3.8	G1, 56 (42.7)		Monthly SP, with directly observed treatment doses at enrollment and then monthly until delivery
Gonza’lez et al., 2014 [23]	SP, 1576 (33.21)	Benin, Gabon, Mozambique, and Tanzania	RCT (NCT00811421)	24.8 ± 6.3	21 ± 7	1. G1, 460 (29) 2. G1-G3, 778 (49) 3. ≥G4, 338 (21)	At least six	IPTp with SP
	MQ, 3169 (66.79)			24.6 ± 6.15	21 ± 7	1. G1, 918 (28.97) 2. G1–G3, 1612 (50.87) 3. ≥G4, 639 (20.16)		IPTp with MQ (15 mg/kg) given once as full dose or IPTp with MQ (15 mg/kg) split over two days
Jagannathan et al., 2018 [24]	SP, 100 (52.36)	Uganda	RCT (NCT02163447)	21.4 ± 3.6	39.3 ± 1.8	1. G1, 35 (35) 2. G2, 33 (33) 3. >G3, 32 (32)	Up to 24	Women: IPTp-SP8w; children: DP every 12 weeks
	Bimonthly DP, 44 (23.04)			23 ± 4.1	39.1 ± 2.6	1. G1, 10 (22.7) 2. G2, 16 (36.4) 3. >G3, 18 (40.9)		Women: IPTp-DP8w; children: DP every 12 weeks
	DPm, 47 (24.61)			23 ± 3.8	40 ± 1.2	1. G1, 10 (21.3) 2. G2, 16 (34) 3. >G3, 21 (44.7)		Women: IPTp-DP8w, children: DP every 4 weeks.
Kayentao et al., 2004 [25]	CQ Px, 394 (33.88)	Mali	RCT (not registered)	19.4 ± 3.2	21.6 ± 3.3	G1, 234 (59.4)	At least 6	Weekly CQ chemoprophylaxis (weekly CQ): treatment dose (25 mg/kg CQ base over 3 days) at first ANC visit, followed by weekly prophylaxis (300 mg CQ base per week)
	CQ, 380 (32.67)			19.1 ± 3.1	21.5 ± 3.1	G1, 243 (64)		Two-dose IPT with CQ (IPT/CQ): treatment doses of 25 mg/kg of CQ base over 3 days at enrollment and again early in third trimester (28–30 weeks gestation)
	SP, 389 (33.45)			19.3 ± 3.3	21.8 ± 2.9	G1, 244 (62.6)		Two-dose IPT with SP (IPT/SP): treatment doses (1500 mg sulfadoxine and 75 mg pyrimethamine)
Kuile et al., 2004 [26]	Bed nets, 381 (48.85)	Kenya	RCT (not registered)	24.974 ± 7.23	24.15 ± 14.66	1. G1–G4, 234 (61.42) 2. >G4, 147 (28.58)	At least 6	Insecticide-treated bed nets
	Control, 399 (51.15)			26.999 ± 6.62	24.4 ± 12.84	1. G1–G4, 229 (57.39) 2. >G4, 170 (42.61)		No nets
Kumar et al., 2020 [27]	Bed nets, 100 (50)	Pakistan	Quasi-experimental study (not registered)	a. ≤25, 39 (39%) b. 26–30, 39 (39%) c. 31 and above, 22 (22%)	NR	NR	At least 6	Long-lasting insecticide-treated bed nets
	Control, 100 (50)			a. ≤25, 22 (22%) b. 26–30, 53 (53%) c. 31 and above, 25 (25%)				Core health workers
Lingani et al., 2023 [28]	SPAZ, 450 (50.11)	Burkina Faso	RCT (PACTR 201808177464681)	26 ± 6	22.4 ± 2	1. G1, 143 (28.8) 2. G2, 103 (20.8) 3. ≥G3, 250 (50.4)	At least 6	Monthly sulfadoxine–pyrimethamine (1500/75 mg) and two grams azithromycin (1 g daily for 2 days) given at second and third trimesters of pregnancy
	SP, 448 (49.89)			25 ± 6	22.4 ± 2	1. G1, 149 (30) 2. G2, 121 (24.4) 3. ≥G3, 226 (45.6)		Monthly sulfadoxine–pyrimethamine (1500/75 mg) (IPTp-SP)
Madanitsa et al., 2023 [29]	SP, 1561 (33.35)	Tanzania, Kenya, and Malawi	RCT (NCT03208179)	24·9 ± 6.1	20.85 ± 3.43	1. G1, 493 (31·6) 2. G2, 373 (23·9) 3. G3 or more, 692 (44·4)	Median (4.3)	Monthly IPTp with sulfadoxine (500 mg)–pyrimethamine (25 mg)
	DPm, 1561 (33.35)			25.1 ± 6.1	20.86 ± 3.4	1. G1, 473 (30·4) 2. G2, 393 (25·2) 3. G3 or more, 691 (44.4)		Monthly IPTp with dihydroartemisinin (40 mg)–piperaquine (320 mg) plus single treatment course of placebo at enrolment
	DPAZ, 1558 (33)			24.9 ± 6	21 ± 3.5	1. G1, 435 (28) 2. G2, 429 (27·6) 3. G3 or more, 689 (44·4)		Monthly IPTp with dihydroartemisinin–piperaquine combined with single dose of azithromycin
Mlugu et al., 2021 [30]	SP, 478 (50)	Tanzania	RCT (PACTR 201612001901313)	26.6 ± 7	21 ± 3	1. G1, 128 (26.8) 2. G2, 105 (22) 3. ≥G3, 245 (51.2)	At least 6	Single dose of three tablets, each containing 500 mg sulfadoxine and 25 mg pyrimethamine
	DPm, 478 (50)			26.8 ± 8	22 ± 3	1. G1, 115 (24.1) 2. G2, 108 (22.6) 3. ≥G3, 255 (53.3)		40 mg dihydroartemisinin and 320 mg piperaquine daily for 3 consecutive days
Moore et al., 2019 [31]	SP, 58 (48.74)	Australia	RCT (not registered)	23 ± 3.041	25 ± 4.56	1.67 (SD 1.52)	At least 6	Single-dose SP (three tablets of 1500 mg sulfadoxine and 75 mg pyrimethamine
	AZ-PQ, 61 (51.24)			23 ± 4.56	25.33 ± 4.56	2 (SD 1.52)		Three daily doses (at 0, 24, and 48 h) of 1 g AZ (film-coated 500 mg tablets) given with 960 mg PQ tetraphosphate (three 320 mg tablets)
Nambozi et al., 2017 [48]	AL, 300 (33.33)	Zambia	RCT (NCT00852423)	20.67 ± 4.47	1. 2nd TM, 150 (50%) 2. 3rd TM, 150 (50%)	1. G1, 100 (33.3) 2. G2, 91 (30.3) 3. ≥G3, 109 (36.3)	At least 3	20 mg artemether and 120 mg lumefantrine per tablet at 4 tablets twice per day over 3 days
	MQAS, 300 (33.33)			20.33 ± 4.47	1. 2nd TM, 150 (50%) 2. 3rd TM, 150 (50%)	1. G1, 107 (35.7) 2. G2, 91 (30.3) 3. ≥G3, 102 (34)		100 mg artesunate and 220 mg mefloquine per tablet at 3 tablets once per day over 3 days
	DP, 300 (33.33)			20.67 ± 4.47	1. 2nd TM, 131 (43.7%) 2. 3rd TM, 169 (56.3%)	1. G1, 94 (31.3) 2. G2, 96 (32) 3. ≥G3, 110 (36.7)		40 mg dihydroartemisinin and 320 mg piperaquine phosphate per tablet, 3 tablets once per day over 3 days
Njagi et al., 2003 [32]	ITN and SP, 198 (26.33)	Kenya	RCT (not registered)	1. G1, 18.4 ± 2.2 2. G2, 21 ± 3.1	1. G1, 20.8 ± 3.5 2. G2, 20.5 ± 3.8	1. G1, 400 (53.19) 2. G2, 352 (46.81)		Rectangular blue or white polyester nets, measuring 190 × 180 × 150 cm dipped into cyfluthrin EW diluted with water to 5 mg/m^2^ concentration and SP
	ITN, 192 (26.06)							Rectangular blue or white polyester nets measuring 190 × 180 × 150 cm dipped into cyfluthrin EW diluted with water to 5 mg/m^2^ concentration
	SP, 183 (24.34)							Sulfadoxine–pyrimethamine tablets
	Control, 175 (23.27)							Identical placebo
Nosten et al., 1993 [33]	MQ, 171 (50.44)	Thailand	RCT (not registered)	26.4 ± 6.2	24.3 ± 3.3	3.7 (SD 2.6)	Up to 24	Mefloquine 500 mg base loading dose followed by 250 mg weekly for 4 weeks and thereafter 125 mg weekly until delivery
	Control, 168 (49.56)			26.5 ± 6.5	24.5 ± 3.4	3.9 (SD 2.8)		Identical placebo
Otuli et al., 2020 [34]	MQ, 156 (50.49)	Republic of Congo	RCT (not registered)	a. ≤18, 39 (12.1%) b. 19–34, 256 (79.2%) c. ≥35, 28 (8.7%)	16 to 28	1. G1, 86 (26.63) 2. G2, 237 (73.37)	At least 6	1 tablet of 250 mg of mefloquine every 8 h at home and with meal
	SP, 153 (49.51)							4 doses of 1500 mg sulfadoxine and 75 mg pyrimethamine taken 4 weeks apart
COSMIC Consortium. 2018 [35]	AL, 2448 (51.95)	Gambia, Benin, and Burkina Faso	RCT (NCT01941264)	25.17 ± 7.014	20.74 ± 3.79	1. G0, 528 (21.75) 2. G1, 412 (16.97) 3. G2, 373 (15.36) 4. G3, 353 (14.54) 5. ≥G4, 769 (31.67)	At least 6	Artemether–lumefantrine
	SP, 2264 (48.05)			24.893 ± 6.9	20.77 ± 3.75	1. G0, 446 (19.7) 2. G1, 412 (18.2) 3. G2, 38,016.78) 4. G3, 348 (15.37) 5. ≥G4, 693 (30.61)		Sulfadoxine–pyrimethamine
Steeke et al., 1996 [36]	CQ Px	Malawi	RCT (not registered)	NR	NR	NR	At least 3	Chloroquine (CQ) treatment dose of 25 mg of base/kg given as divided dose over two days, followed by 300 mg weekly
	CQ							CQ treatment dose of 25 mg of base/kg given as divided dose over two days and repeated every four weeks
	Weekly CQ							CQ, 300 mg of base weekly
	MQ							Mefloquine (MQ) treatment dose of 750 mg as a single dose followed by 250 mg weekly
Browne et al., 2001 [37]	Bed nets, 1033 (52.68)	Ghana	RCT (not registered)	NR	1. 1st TM, 20 (1.9%) 2. 2nd TM, 388 (37.6%) 3. 3rd TM, 625 (60.5%)	1. G1, 204 (19.7) 2. G2, 168 (16.3) 3. ≥G3, 661 (64)	At least 6	Insecticide-treated bed nets
	Control, 928 (47.32)				1. 1st TM, 18 (1.9%) 2. 2nd TM, 350 (37.7%) 3. 3rd TM, 560 (60.4%)	1. G1, 202 (21.8) 2. G2, 162 (17.5) 3. ≥G3, 564 (60.7)		No nets
Kajubi et al., 2017 [38]	EFV, 27 (30.68)	Uganda	RCT (NCT02163447)	30 ± 6.25	12 to 28	NR	Up to 9	EFV-based ART, standard single-tablet regimen of EFV (600 mg), tenofovir disoproxil fumarate (300 mg), and lamivudine (300 mg) once daily
	Control, 31 (35.23)			23 ± 3.25				DHA–piperaquine, standard dose (3 tablets (40 mg DHA and 320 mg piperaquine) once daily) for 3 consecutive days with or without food
	Non pregnant Control, 30 (30.09)			24 ± 3.25	NA	NA		DHA–piperaquine, standard dose (3 tablets (40 mg DHA and 320 mg piperaquine) once daily) for 3 consecutive days with or without food
Roh et al., 2022 [39]	LLINs, 4207 (39)	Multicenter	Quasi-experimental study (not registered)	24.3 ± 1	NR	25.8 (SD 10.8)	At least 9	Long-lasting insecticidal nets
	PBO LLINs, 4473 (43.31)			24.4 ± 1.9		23.6 (SD 9.4)		Piperonyl butoxide long-lasting insecticidal nets
	LLINs + PBO LLINs, 1828 (17.7)			23.6 ± 0.6		32.1 (SD 4.6)		Long-lasting insecticidal nets + piperonyl butoxide
Akinyotu et al., 2018 [40]	MQ, 64 (48.85)	Nigeria	RCT (NCT02524444)	34.67 ± 4.37	At least 16	a. <20, 24 (41) b. 20–24, 30 (56) c. >24, 10 (53)	At least 4	Mefloquine (synthetic 4-quinoline methanol derivative related to quinine) administered in three doses of 250 mg at 4-week intervals
	SP, 67 (51.15)			32.12 ± 5.66		a. <20, 34 (59) b. 20–24, 24 (44) c. >24, 9 (47)		Sulfadoxine–pyrimethamine, comprising 500 mg sulfadoxine and 25 mg pyrimethamine, also administered in three doses with 4-week intervals
Akinyotu et al., 2019 [41]	SPAZ, 60 (48.78)	Nigeria	RCT (not registered)	33.2 ± 4.92	a. <20, 19 (31.7%) b. 20–24, 35 (58.3%) c. >24, 6 (10%)	1. G0, 6 (10) 2. G1, 21 (35) 3. G2, 27 (45) 4. ≥G3, 6 (10)	At least 6	Monthly doses of SP (consisting of three tablets each containing 500 mg/25 mg) administered for 3 months as IPT-p with daily dose of AZ (consisting of one 500 mg tablet) administered for 3 d as IPT-p in HIV-positive pregnant women
	SP, 63 (51.22)			32.17 ± 5.64	a. <20, 32 (50.8%) b. 20–24, 22 (34.9%) c. >24, 9 (14.3%)	1. G0, 19 (30.2) 2. G1, 21 (33.3) 3. G2, 14 (22.2) 4. ≥G3, 9 (14.3)		Monthly doses of SP (consisting of three tablets each containing 500 mg/25 mg) administered for 3 months as IPT-p
Barsosio et al., 2024 [42]	DPm and CTX, 448 (49.56)	Malawi	RCT (NCT04158713)	29.2 ± 5.6	22 ± 3.7	1. G1, 32 (7) 2. G2, 88 (20) 3. ≥G3, 328 (73)	At least 9	Daily Co-trimoxazole combined with monthly IPTp with active dihydroartemisinin–piperaquine
	CTX, 456 (50.46)			29.2 ± 5.7	22 ± 3.8	1. G1, 37 (8) 2. G2, 91 (20) 3. ≥G3, 328 (72)		Co-trimoxazole combined with monthly identical placebo
Gonza’lez et al., 2014 [43]	MQ, 534 (49.86)	Kenya, Tanzania, and Mozambique	RCT (NCT 00811421)	26.8 ± 5.8	21 ± 8	1. G1, 57 (11) 2. G1–G3, 341 (64) 3. ≥G4, 136 (25)	At least 9	CTX (fixed combination of 800 mg trimethoprim and 160 mg sulfamethoxazole/tablet) plus IPTp-MQ (250 mg of MQ base/tablet)
	Control, 537 (50.14)			26.6 ± 5.4	21 ± 7	1. G1, 51 (9) 2. G1–G3, 363 (68) 3. ≥G4, 122 (23)		CTX plus IPTp-placebo (identical to MQ tablets in shape and color)
Manirakiza et al., 2021 [44]	CTX, 47.77)	Central African Republic	RCT (NCT01746199)	27.167 ± 6.78	21 ± 4.521	G1, 7 (8)	At least 6	One daily tablet containing 160 mg of trimethoprim and 800 mg of sulfamethoxazole) was administered from 16 weeks until the end of pregnancy
	SP, 98 (52.23)			29.67 ± 6.781	21.67 ± 6.02	G1, 9 (9)		Three doses of SP-IPTp (1500 mg sulfadoxine and 75 mg pyrimethamine per dose) given under directly observed administration at one-month intervals from 16 weeks gestation
Ndam et al., 2013 [45]	CTX, 152 (48.72)	Benin	RCT (NCT00970879)	At least 18	38.3 ± 1.82	NR	At least 7	CTX at daily dose of 800 mg sulfamethoxazole and 160 mg trimethoprim
	MQ, 160 (51.28)				38.46 ± 1.57			15 mg/kg MQ (Cipla, Mumbai, India) with rich-fat collation under direct observation
Olofin et al., 2014 [46]	No Multivitamins, 522 (24.86)	Tanzania	RCT (not registered)	25.3 ± 4.8	20.3 ± 3.6	1. G0, 137 (26.3) 2. G1–3, 299 (57.3) 3. >G3, 86 (16.4)	At least 6	Identical placebo
	Multivitamins, 528 (25.14)			25.4 ± 4.7	20.4 ± 3.2	1. G0, 142 (26.9) 2. G1–3, 318 (60.3) 3. >G3, 68 (12.8)		(20 mg vitamin B1, 20 mg B2, 25 mg B6, 100 mg niacin, 50 mg B12, 500 mg C, 30 mg E, and 800 mg folic acid) and Vitamin A
	No Vitamin A, 521 (24.81)			25.4 ± 4.8	20.5 ± 3.3	1. G0, 141 (27.1) 2. G1–3, 306 (58.7) 3. >G3, 74 (14.2)		(20 mg vitamin B1, 20 mg B2, 25 mg B6, 100 mg niacin, 50 mg B12, 500 mg C, 30 mg E, and 800 mg folic acid)
	Vitamin A, 529 (25.19)			25.3 ± 4.8	20.3 ± 3.4	1. G0, 139 (26.2) 2. G1–3, 311 (58.8) 3. >G3, 79 (15)		Vitamin A alone (30 mg b-carotene with 5000 IU preformed vitamin A)
Natureeba et al., 2017 [47]	TMP-SMX, 100 (50)	Uganda	RCT (NCT02282293)	30.3 ± 5.8	19.2 ± 4.1	1. G1, 5 (5) 2. G2, 13 (13) 3. ≥G3, 82 (82)	At least 7	Daily trimethoprim–sulfamethoxazole (160 mg/800 mg)
	TMP-SMX and Monthly DP, 100 (50)			29.8 ± 6.8	19.9 ± 4.5	1. G1, 13 (13) 2. G2, 12 (12) 3. ≥G3, 75 (75)		Daily trimethoprim–sulfamethoxazole (160 mg/800 mg) and DP (40 mg dihydroartemisinin plus 320 mg piperaquine

Abbreviations: RCT = randomized controlled trial; SD = standard deviation; NR = not reported; NA = not applicable; SST = single screening and treatment; IST = intermittent screening and treatment; IPTp = intermittent preventive treatment during pregnancy; DPm = monthly dihydroartemisinin; MQ = mefloquine; SP = sulfadoxine and pyrimethamine; AQ = amodiaquine; SPAQ = sulfadoxine and pyrimethamine plus amodiaquine; VA = vitamin A; CQ Px = prophylactic chloroquine; SPAZ = sulfadoxine and pyrimethamine plus Azithromycin; DPAZ = dihydroartemisinin and azithromycin; AZ-PQ = azithromycin and piperaquine; MQAS = mefloquine–artesunate; ITN = insecticide-treated net; AL = artemether–lumefantrine; EFV = efavirenz; LLINs = long-lasting insecticide-treated bed nets; CTX = Co-trimoxazole; TMP-SMX = trimethoprim-sulfa-methoxasole.

**Table 2:** Summary of included studies.

Study ID	Diagnostic Tools	Inclusion Criteria	Primary Endpoints	Conclusion
Ahmed et al., 2019 [14]	Quantitative PCR [qPCR], nested PCR, and loop-mediated isothermal amplification [LAMP] (Eiken Chemical Company, Japan)	1. Between 16 May 2013, and 21 April 2016 2. Pregnant women of any gravity 3. Viable pregnancy between 16 and 30 weeks gestation 4. Had given written informed consent	1. Malaria infections 2. Adverse events	“IST was associated with a lower prevalence of malaria than SST at delivery, but the prevalence of malaria in this group was also lower at enrolment, interpreting the effect of IST as challenging. Further studies with highly sensitive malaria rapid diagnostic tests should be considered. Monthly IPT with dihydroartemisinin–piperaquine is a promising alternative to SST in areas in the Asia-Pacific region with moderate or high malaria transmission”.
Briand et al., 2009 [15]	Thick and thin blood smears stained with Giemsa stain	1. In Benin from July 2005 through April 2008 2. Women of all gravidities of 16–28 weeks gestation 3. No history of neurologic or psychiatric disorder 4. Followed for at least 5.27 months	1. Malaria infections 2. LBW 3. Safety and adverse events	“MQ proved to be highly efficacious—clinically and parasitologically—for use as IPTp. However, its low tolerability might impair its effectiveness and requires further investigations”.
Clerk et al., 2008 [16]	Thick blood film	1. From June 2004 to February 2007 2. Highly endemic area of malaria 3. Availability for follow-up during pregnancy 4. Willingness to comply with study procedures	1. Malarial infections 2. LBW 3. Safety and adverse events	“The effects of IPTp with AQ or SPAQ on maternal anaemia and LBW were comparable to the effects of IPTp with SP; however, IPTp regimens that contain AQ are unlikely to be useful as an alternative to IPTp with SP in Ghana because of a high frequency of associated adverse events”.
Cox et al., 2005 [17]	Microscopic examination of Giemsa-stained thick blood films	1. From March to June 2001 2. Primigravid pregnant women 3. Resident within study area 4. In good health and less than 24 weeks pregnant 5. Followed up for maximum 16 weeks	1. Malarial infections 2. LBW	“The data suggest that the reduction in the levels of anti-VSACSA antibodies to the known placental malaria isolate may reflect reduced intensity or duration of placental parasitemia in women receiving vitamin A supplementation. These observations are of potential public health significance and deserve further investigation”.
Darling et al., 2017 [18]	Histopathology and polymerase chain reaction (PCR)	1. Participants in first trimester of pregnancy 2. Primigravida or secundigravida 3. Human immunodeficiency virus (HIV)-negative 4. Intending to stay in Dar es Salaam for at least 6 weeks after delivery 5. Followed up for at least 10 months	1. Malarial infections 2. LBW 3. Safety and adverse events	“No safety concerns were identified. We recommend additional studies in other geographic locations to confirm these findings”.
Desai et al., 2015 [19]	PCR	1. HIV-negative pregnant women 2. Between 16 and 32 weeks gestation 3. Viable pregnancy 4. No history of receiving IPTp-SP during pregnancy	Malarial infections	“At the current levels of RDT sensitivity, ISTp is not a suitable alternative to IPTp-SP in the context of high SP resistance and malaria transmission. However, DP is a promising alternative drug to replace SP for IPTp. The efficacy, operational feasibility, and cost-effectiveness of IPTp-DP should be investigated further”.
Divala et al., 2018 [20]	Histopathology, molecular results, or PCR	1. Pregnant women in their first or second pregnancy 2. Before 27th week of gestation 3. Not yet taken routine SP IPTp 4. Hoped to remain in area until 14 weeks after delivery	1. Malaria infection 2. Maternal anemia	“Chloroquine administered as IPTp did not provide better protection from malaria and related adverse effects than SP-IPTp in this setting of high SP-resistance. Protocol-specified adjusted analyses suggest that chloroquine chemoprophylaxis may provide benefit in protecting against malaria during pregnancy”.
Dolan et al., 1993 [21]	Blood taken by finger-prick for thick and thin films	Pregnant women given either permethrin-impregnated bed net (PIB), an untreated bed net (NIB), or no study bed net	1. Malaria infection 2. LBW	“PIB or FNIB reduce the adverse effects of malaria in pregnancy on the mother, and may also reduce subsequent infant morbidity and mortality”.
Filler et al., 2006 (Non-HIV) [22]	Thick blood smears stained with Giemsa	1. Clinic patients seeking ANC 2. Women in their first and second pregnancies 3. Between 16 and 28 weeks of gestation 4. Had given informed consent	1. Malaria infection 2. Safety and adverse events	“In HIV-positive pregnant women, monthly SP IPTp is more efficacious than a 2-dose regimen in preventing placental malaria. The study also demonstrates the continued efficacy of SP for the prevention of placental malaria, even in the face of its decreasing efficacy for the treatment of malaria in children. In areas with intense transmission of falciparum malaria and a high prevalence of HIV infection, monthly SP IPTp should be adopted”.
Filler et al., 2006 (HIV) [22]	Thick blood smears stained with Giemsa	1. Clinic patients seeking ANC 2. Women in their first and second pregnancies 3. Between 16 and 28 weeks of gestation 4. Had given informed consent	1. Malaria infection 2. Safety and adverse events	“In HIV-positive pregnant women, monthly SP IPTp is more efficacious than a 2-dose regimen in preventing placental malaria. The study also demonstrates the continued efficacy of SP for the prevention of placental malaria, even in the face of its decreasing efficacy for the treatment of malaria in children. In areas with intense transmission of falciparum malaria and a high prevalence of HIV infection, monthly SP IPTp should be adopted”.
Gonza’lez et al., 2014 [23]	Thick and thin blood films stained	1. Pregnant women of all gravidities attending ANC clinic for first time 2. Had not received IPTp during current pregnancy 3. Gestational age < 28 weeks 4. Negative HIV testing at recruitment 5. Absence of history of allergy to sulfa drugs or MQ	1. Malaria infection 2. LBW 3. Safety and adverse drug reactions	“Women taking MQ IPTp (15 mg/kg) in the context of long-lasting insecticide-treated nets had similar prevalence rates of low birth weight as those taking SP IPTp. MQ recipients had less clinical malaria than SP recipients, and the pregnancy outcomes and safety profile were similar. MQ had poorer tolerability even when splitting the dose over two days. These results do not support a change in the current IPTp policy”.
Jagannathan et al., 2018 [24]	Blood collected for thick blood smear	1. From June 2014 through May 2017 2. Area of historically high malaria transmission intensity 3. Pregnant women of at least 16 weeks gestation 4. Negative HIV testing at recruitment	1. Malaria infection 2. Maternal anemia 3. Safety and adverse drug reactions	“Contrary to our hypothesis, preventing malaria in pregnancy with IPTp-DP in the context of chemoprevention with DP during infancy does not lead to a reduced incidence of malaria in childhood; in this setting, it may be associated with an increased incidence of malaria in females. Future studies are needed to better understand the biological mechanisms of in utero drug exposure on drug metabolism and how this may affect the dosing of antimalarial drugs for treatment and prevention during infancy”.
Kayentao et al., 2004 [25]	Thick blood films stained with Giemsa	1. Women with first or second pregnancy and between 16 and 26 weeks of gestation 2. Had given written informed consent	Malaria infection	“In Mali, IPT with SP appears more efficacious than weekly chloroquine chemoprophylaxis in preventing malaria during pregnancy. These data support World Health Organization recommendations to administer at least 2 doses of IPT during pregnancy. In intensely seasonal transmission settings in Mali, 12 doses may be required to prevent placental reinfection prior to delivery”.
Kuile et al., 2004 [26]	Malaria thick and thin blood smears	1. Pregnant women who had parasitemia 2. Documented fever or patients with history of fever within previous 48 h treated with SP 3. At least 16 weeks gestation 4. Negative HIV testing at recruitment	1. Malaria infection 2. Maternal anemia 3. Safety and adverse events	“In areas of intense perennial malaria transmission, permethrin-treated bed nets reduce the adverse effect of malaria during the first four pregnancies”.
Kumar et al., 2020 [27]	NR	Pregnant women given either permethrin-impregnated bed net (PIB), untreated bed net (NIB), or no study bed net	Incidence of malaria infections	“Results proved that health education could be an effective intervention for improving knowledge and usage of LLINs among pregnant women for the prevention of malaria. Such educational interventions have a positive potential to be implemented at a larger scale by incorporating them into routine health sessions provided by health workers”.
Lingani et al., 2023 [28]	Thick and thin blood smears stained with 5% Giemsa for 30 min	1. Age of 16–35 years 2. A gestational age of 12–24 weeks 3. Negative HIV-testing at recruitment 4. Willingness to adhere to study protocol and signed informed consent	1. Malaria infection 2. LBW 3. Safety and adverse events	“Adequate prevention regimen with monthly sulfadoxine-pyrimethamine given to all pregnant women has been proven to reduce the risk of LBW in malaria-endemic areas. Adding azithromycin to the regimen does not offer further benefits, as long as women receive a malaria prevention regimen early enough during pregnancy”.
Madanitsa et al., 2023 [29]	Malaria microscopy, quantitative PCR (qPCR), and targeted next-generation sequencing for molecular markers	1. From 29 March 2018 to 5 July 2019 2. Women of any age with viable singleton pregnancy 3. Between 16 weeks and 28 weeks gestation confirmed by the US 4. Willingness to adhere to study protocol and signed informed consent	1. Malaria infection 2. LBW 3. Safety and adverse events	“Monthly IPTp with dihydroartemisinin–piperaquine did not improve pregnancy outcomes, and the addition of a single course of azithromycin did not enhance the effect of monthly IPTp with dihydroartemisinin–piperaquine. Trials that combine sulfadoxine-pyrimethamine and dihydroartemisinin–piperaquine for IPTp should be considered”.
Mlugu et al., 2021 [30]	RDT, microscopy, and PCR	1. HIV-negative, age 16 years or older 2. Malaria-negative (RDT) 3. Gestational age of ≥13 weeks 4. Willing and able to give informed consent	1. Malaria infection 2. LBW 3. Safety and adverse events	“However, the prevalence of LBW (4.6% versus 9.6%, *p* = 0.003) was significantly lower in IPTp-DHP compared to IPTp-SP. We report superior protective efficacy of monthly IPTp-DHP against malaria in pregnancy and LBW than IPTp-SP”.
Moore et al., 2019 [31]	Thick/thin blood smears prepared for microscopy	1. Pregnant women between 14 and 32 weeks of gestation 2. Not taken any study drugs in previous 28 days 3. No history of allergy to study drugs 4. Willing and able to give informed consent	1. Malaria infection 2. Maternal anemia 3. Safety and adverse events	“Further assessment of AZ-PQ (including alternative total dosing of AZ, with a focus on tolerability) should be undertaken in a variety of settings in which malaria is endemic, to ensure that this therapy would be accepted as an alternative to SP”.
Nambozi et al., 2017 [48]	Giemsa-stained thick and thin blood films	Pregnant women with 20 weeks median gestational age	Malaria infection	“As new infections can be prevented by a long-acting partner drug to the artemisinins, DHAPQ should be preferred in places such as Nchelenge district where transmission is intense while in areas of low transmission intensity AL or MQAS may be used”.
Njagi et al., 2003 [32]	Giemsa-stained thick and thin blood films	1. Pregnant women estimated at gestational age between 12 and 24 weeks 2. Had given informed consent	Malaria infection	“It was concluded that malaria is a major cause of anemia in primigravidae but that other causes play a more significant role in secundigravidae, and that intermittent treatment with SP or use of ITNs benefits primigravidae more than secundigravidae”.
Nosten et al., 1993 [33]	Thick blood film stained with Giemsa stain and examined	Pregnant women of at least 20 weeks gestation	1. Malaria infection 2. Maternal anemia 3. Safety and adverse events	“Mefloquine is safe and effective for antimalarial prophylaxis in the second half of pregnancy”.
Otuli et al., 2020 [34]	Finger-prick performed on finger pulp and 5 μL blood dropped and spread on slide	1. From 15 May to 30 November 2019 2. High rate of pregnant women attending ANC 3. Between 16 and 28 gestational weeks 4. Not taking IPTp during current pregnancy 5. Willing and able to give informed consent	1. Malaria infection 2. Safety and adverse events	“Splitting dose and intake with a meal increased mefloquine tolerability while keeping its efficacy higher compared to sulfadoxine–pyrimethamine. Intermittent preventive treatment during pregnancy using mefloquine reduces the risk of placental malaria, maternal peripheral parasitemia, and low birth weight, compared to sulfadoxine–pyrimethamine. Thus, mefloquine is a good alternative to intermittent preventive treatment in pregnancy”.
COSMIC Consortium. 2018 [35]	Giemsa-stained thick blood films	1. Pregnant women in second or third trimester 2. Attending first ANC 3. Willing and able to give informed consent	1. Malaria infection 2. Maternal anemia 3. Safety and adverse events	“Adding CSST to existing IPTp-SP strategies did not reduce malaria in pregnancy. Increasing the number of IPTp-SP doses given during pregnancy is a priority”.
Steeke et al., 1996 [36]	Thick blood smears, hematocrit, and serum for anti-malarial antibody testing	1. Consecutive women of any parity 2. During 1987–1990 3. Pregnant women in area of high malaria endemicity 4. Willing and able to give informed consent	1. Malaria infection 2. LBW	“When evaluating antenatal care programs, health policymakers must consider providing an effective preventive drug (either MQ or other drugs identified in additional studies, e.g., sulfa-pyrimeth-amine compounds) to prevent low birth weight and its consequences”.
Browne et al., 2001 [37]	Giemsa-stained thick and thin blood films	1. Pregnant women 2. Special focus on primigravidae and secundigravidae 3. Willing and able to give informed consent	1. Malaria infection 2. LBW 3. Maternal anemia	“Chloroquine use in pregnancy was low and comparable in both groups. Implications of findings for malaria control in pregnancy and further research are discussed”.
Kajubi et al., 2017 [38]	Giemsa-stained thick and thin blood films	1. HIV-negative pregnant women 2. HIV-positive pregnant women on EFV-based ART 3. HIV-negative non-pregnant women 4. Between 12 and 28 weeks gestation 5. Willing and able to give informed consent	Plasma concentration–time profile	“Exposure to DHA and piperaquine were lower among pregnant women and particularly in women on efavirenz, suggesting a need for dose modifications. The study of modified dosing strategies for these populations is urgently needed”.
Roh et al., 2022 [39]	NR	1. Between March 2017 and March 2018 2. Health facilities from each HSD 3. Government-operated 4. Included maternity ward 5. Located >5 km from neighboring HSD 6. had a mean delivery rate of >200/year	1. LBW incidence 2. Stillbirth incidence	“In this region of Uganda, where pyrethroid resistance is high, this study found that a mass LLIN campaign was associated with reduced stillbirth incidence. Effects of the campaign were greatest for women who would”.
Akinyotu et al., 2018 [40]	Thick and thin blood smears	1. Between 1 January and 31 August 2016 2. HIV-positive pregnant women 3. At least 16 weeks of gestation 4. Followed up for at least four months	Malaria infection	“Outcomes following prophylactic use of mefloquine for intermittent preventive therapy for malaria among pregnant women with HIV were comparable to sulphadoxine–pyrimethamine treatment; mefloquine is a feasible alternative therapy”.
Akinyotu et al., 2019 [41]	Thick and thin blood smears	1. Between 1 January and 31 August 2016 2. HIV-positive patients 3. At least 16 weeks of gestation 4. No history of AZ or SP use for 4 weeks prior to recruitment	1. Malaria infections 2. LBW 3. Safety and adverse events	“The findings suggest that AZ is comparable to SP in malaria prevention and safety in HIV-positive pregnant women”.
Barsosio et al., 2024 [42]	Microscopy, PCR, and blood tests	1. From 11 November 2019 to 3 August 2021 2. HIV-positive pregnant patients 3. Between 16 weeks and 28 weeks gestation 4. Willingness to give informed consent	1. Malaria infections 2. LBW 3. Safety and adverse events	“Addition of monthly intermittent preventive treatment with dihydroartemisinin–piperaquine to the standard of care with daily unsupervised co-trimoxazole in areas of high antifolate resistance substantially improves malaria chemoprevention in pregnant women living with HIV on dolutegravir-based cART and should be considered for policy”.
Gonza’lez et al., 2014 [43]	Microscopy on Giemsa-stained blood films	1. From March 2010 to April 2012 2. HIV-positive pregnant patients 3. Gestational age ≤ 28 weeks 4. Absence of history of allergy to sulfa drugs or MQ 5. Willingness to give informed consent	1. Malaria infections 2. Maternal anemia 3. Safety and adverse events	“An effective antimalarial added to CTXp and LLITNs in HIV-infected pregnant women can improve malaria prevention, as well as maternal health through reduction in hospital admissions. However, MQ was not well tolerated, limiting its potential for IPTp and indicating the need to find alternatives with better tolerability to reduce malaria in this particularly vulnerable group. MQ was associated with an increased risk of mother-to-child transmission of HIV, which warrants a better understanding of the pharmacological interactions between antimalarials and antiretroviral drugs”.
Manirakiza et al., 2021 [44]	PCR	1. HIV-positive pregnant patients 2. Between 16 and 28 weeks of gestation 3. CD4+ count ≥ 350 cells/mm^3^ 4. Willingness to give informed consent	1. Malaria infections 2. Safety and adverse events	“Although our results do not allow us to conclude that CTX is more effective, drug safety and good compliance among women with this treatment favor its widespread use among HIV-infected pregnant women, as currently recommended by WHO”.
Ndam et al., 2013 [45]	Blood samples collected in EDTA tubes then PCR	1. HIV-positive pregnant women 2. Between 16 and 28 weeks of gestation 3. Willingness to give informed consent	1. Malaria infections 2. Maternal anemia	“CTX alone provided adequate protection against malaria in HIV-infected pregnant women, although MQ-IPTp showed higher efficacy against placental infection. Although more frequently associated with dizziness and vomiting, MQ-IPTp may be an effective alternative given concerns about parasite resistance to CTX”.
Olofin et al., 2014 [46]	Thick and thin films stained with 5% Giemsa solution and examined	1. From April 1995 until August 2003 2. HIV-positive pregnant patients 3. Between 16 and 28 weeks of gestation 4. Willingness to give informed consent	1. Malaria infections 2. Maternal anemia 3. Safety and adverse events	“Multivitamin supplements have been previously shown to reduce HIV disease progression among HIV-infected women, and consistent with that, these supplements protected against development of symptomatic malaria. The clinical significance of the increased risk of malaria parasitemia among supplemented women deserves further research, however. Preventive measures for malaria are warranted as part of an integrated approach to the care of HIV-infected individuals exposed to malaria”.
Natureeba et al., 2017 [47]	Blood smears stained with 2% Giemsa and read by experienced laboratory technologist	1. Women ≥16 years of age 2. Positive for HIV-1 3. Between 16 and 28 weeks of gestation 4. Willingness to give informed consent	1. Malaria infections 2. Safety and adverse events	“Among HIV-infected pregnant women in the setting of indoor residual spraying of insecticide, adding monthly DP to daily TMP-SMX did not reduce the risk of placental or maternal malaria or improve birth outcomes”.

Abbreviations: RCT = randomized controlled trial; SD = standard deviation; NR = not reported; NA = not applicable; SST = single screening and treatment; IST = intermittent screening and treatment; IPTp = intermittent preventive treatment during pregnancy; DPm = monthly dihydroartemisinin; MQ = mefloquine; SP = sulfadoxine and pyrimethamine; AQ = amodiaquine; SPAQ = sulfadoxine and pyrimethamine plus amodiaquine; VA = vitamin A; CQ Px = prophylactic chloroquine; SPAZ = sulfadoxine and pyrimethamine plus azithromycin; DPAZ = dihydroartemisinin and azithromycin; AZ-PQ = azithromycin and piperaquine; MQAS = mefloquine–artesunate; ITN = insecticide-treated net; AL = artemether–lumefantrine; EFV = efavirenz; LLINs = long-lasting insecticide-treated bed nets; CTX = Co-trimoxazole; TMP-SMX = trimethoprim-sulfa-methoxasole.

## Data Availability

Data are available from the corresponding author upon reasonable request.

## Supplementary Materials

The following supporting information can be downloaded at: https://www.mdpi.com/article/10.3390/jcm14103396/s1, Table S1: Detailed search strategy for retrieved databases. Table S2: Quality assessment of included quasi-experimental studies.; Figure S1: Incidence of maternal anemia at delivery in pregnant women with HIV. (A) Network graph showing direct evidence between evaluated interventions. (B) Forest plot comparing all interventions. (C) League table representing network meta-analysis estimates for all interventions’ comparisons.; Figure S2: Incidence of low birth weight in pregnant women with HIV. (A) Network graph showing direct evidence between evaluated interventions. (B) Forest plot comparing all interventions. (C) League table representing network meta-analysis estimates for all interventions’ comparisons.; Figure S3: Incidence of dizziness in pregnant women with HIV. (A) Network graph showing direct evidence between evaluated interventions. (B) Forest plot comparing all interventions. (C) League table representing network meta-analysis estimates for all interventions’ comparisons.; Figure S4: Incidence of headache in pregnant women with HIV. (A) Network graph showing direct evidence between evaluated interventions. (B) Forest plot comparing all interventions. (C) League table representing network meta-analysis estimates for all interventions’ comparisons.; Figure S5: Incidence of vomiting in pregnant women with HIV. (A) Network graph showing direct evidence between evaluated interventions. (B) Forest plot comparing all interventions. (C) League table representing network meta-analysis estimates for all interventions’ comparisons.; Figure S6: Incidence of preterm births in pregnant women with HIV. (A) Network graph showing direct evidence between evaluated interventions. (B) Forest plot comparing all interventions. (C) League table representing network meta-analysis estimates for all interventions’ comparisons.; Figure S7: Incidence of stillbirths in pregnant women with HIV. (A) Network graph showing direct evidence between evaluated interventions. (B) Forest plot comparing all interventions. (C) League table representing network meta-analysis estimates for all interventions’ comparisons.; Figure S8: Incidence of maternal anemia at delivery in pregnant women without HIV. (A) Network graph showing direct evidence between evaluated interventions. (B) Forest plot comparing all interventions. (C) League table representing network meta-analysis estimates for all interventions’ comparisons.; Figure S9: Incidence of low birth weight in pregnant women without HIV. (A) Network graph showing direct evidence between evaluated interventions. (B) Forest plot comparing all interventions. (C) League table representing network meta-analysis estimates for all interventions’ comparisons.; Figure S10: Incidence of nausea in pregnant women without HIV. (A) Network graph showing direct evidence between evaluated interventions. (B) Forest plot comparing all interventions. (C) League table representing network meta-analysis estimates for all interventions’ comparisons.; Figure S11: Incidence of vomiting in pregnant women without HIV. (A) Network graph showing direct evidence between evaluated interventions. (B) Forest plot comparing all interventions. (C) League table representing network meta-analysis estimates for all interventions’ comparisons.; Figure S12: Incidence of headache in pregnant women without HIV. (A) Network graph showing direct evidence between evaluated interventions. (B) Forest plot comparing all interventions. (C) League table representing network meta-analysis estimates for all interventions’ comparisons.; Figure S13: Incidence of stillbirths in pregnant women without HIV. (A) Network graph showing direct evidence between evaluated interventions. (B) Forest plot comparing all interventions. (C) League table representing network meta-analysis estimates for all interventions’ comparisons.

## Abbreviations

SST	Single screening and treatment
IST	Intermittent screening and treatment
IPTp	Intermittent preventive treatment during pregnancy
DPm	Monthly dihydroartemisinin
MQ	Mefloquine
SP	Sulfadoxine and pyrimethamine
AQ	Amodiaquine
SPAQ	Sulfadoxine and pyrimethamine plus amodiaquine
VA	Vitamin A
CQ Px	Prophylactic chloroquine
SPAZ	Sulfadoxine and pyrimethamine plus azithromycin
DPAZ	Dihydroartemisinin and azithromycin
AZ-PQ or AZP	Azithromycin and piperaquine
MQAS	Mefloquine–artesunate
ITN	Insecticide-treated Nets
AL	Artemether–lumefantrine
EFV	Efavirenz
LLINs	Long-lasting insecticide-treated bed nets
CTX	Co-trimoxazole
TMP-SMX	Trimethoprim-sulfa-methoxazole
AZ	Azithromycin
