# Supplementary material for: Evaluating the Safety and Efficacy of Malaria Preventive Measures in Pregnant Women with a Focus on HIV Status: A Systematic Review and Network Meta-Analysis
Source: J Clin Med. 2025 May 13;14(10):3396. doi: 10.3390/jcm14103396 (PMC12112236; doi:10.3390/jcm14103396)
Supplement: Supplementary file 1 [file jcm-14-03396-s001.zip › Figure S11.pdf]

A

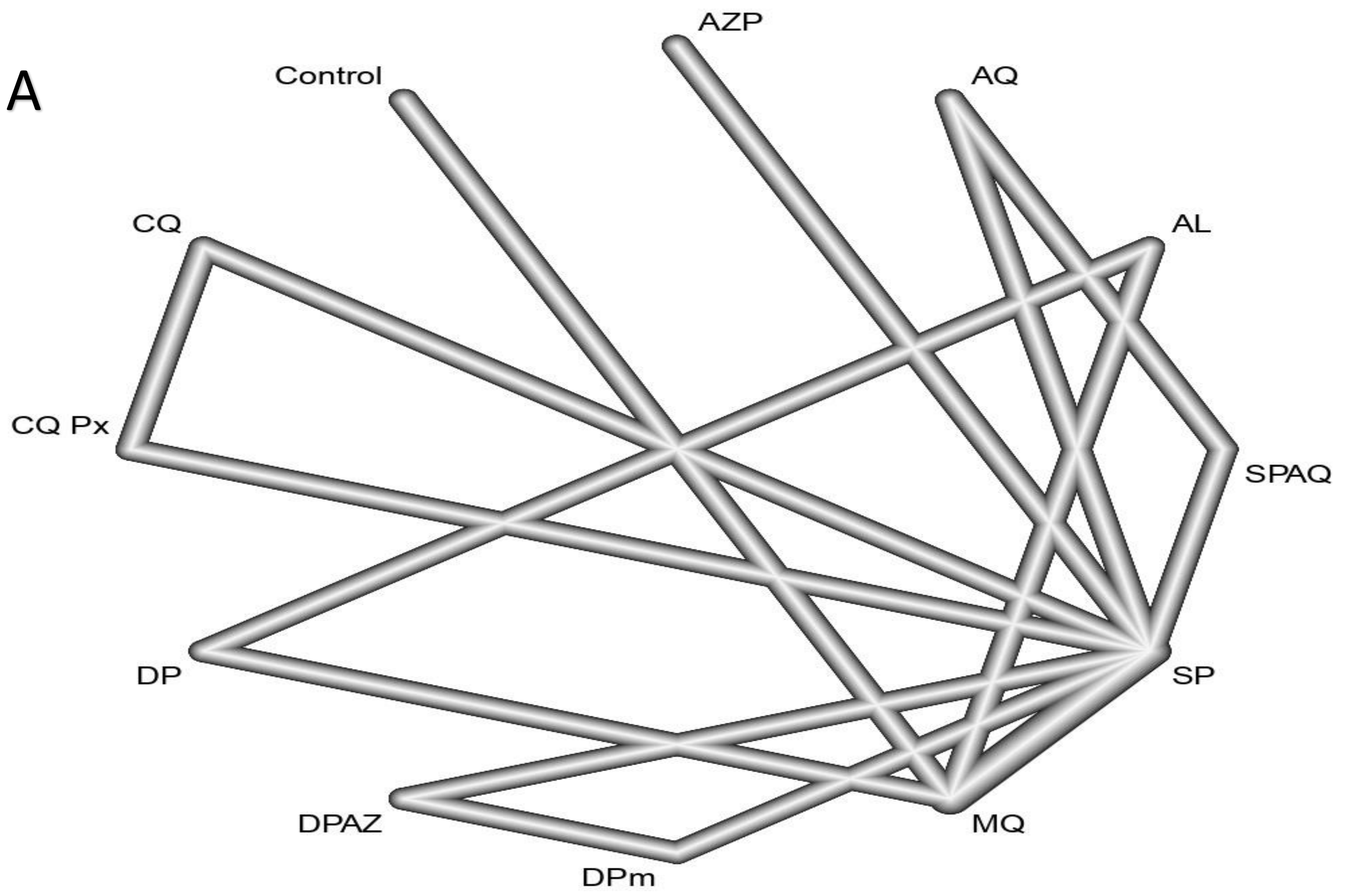

B

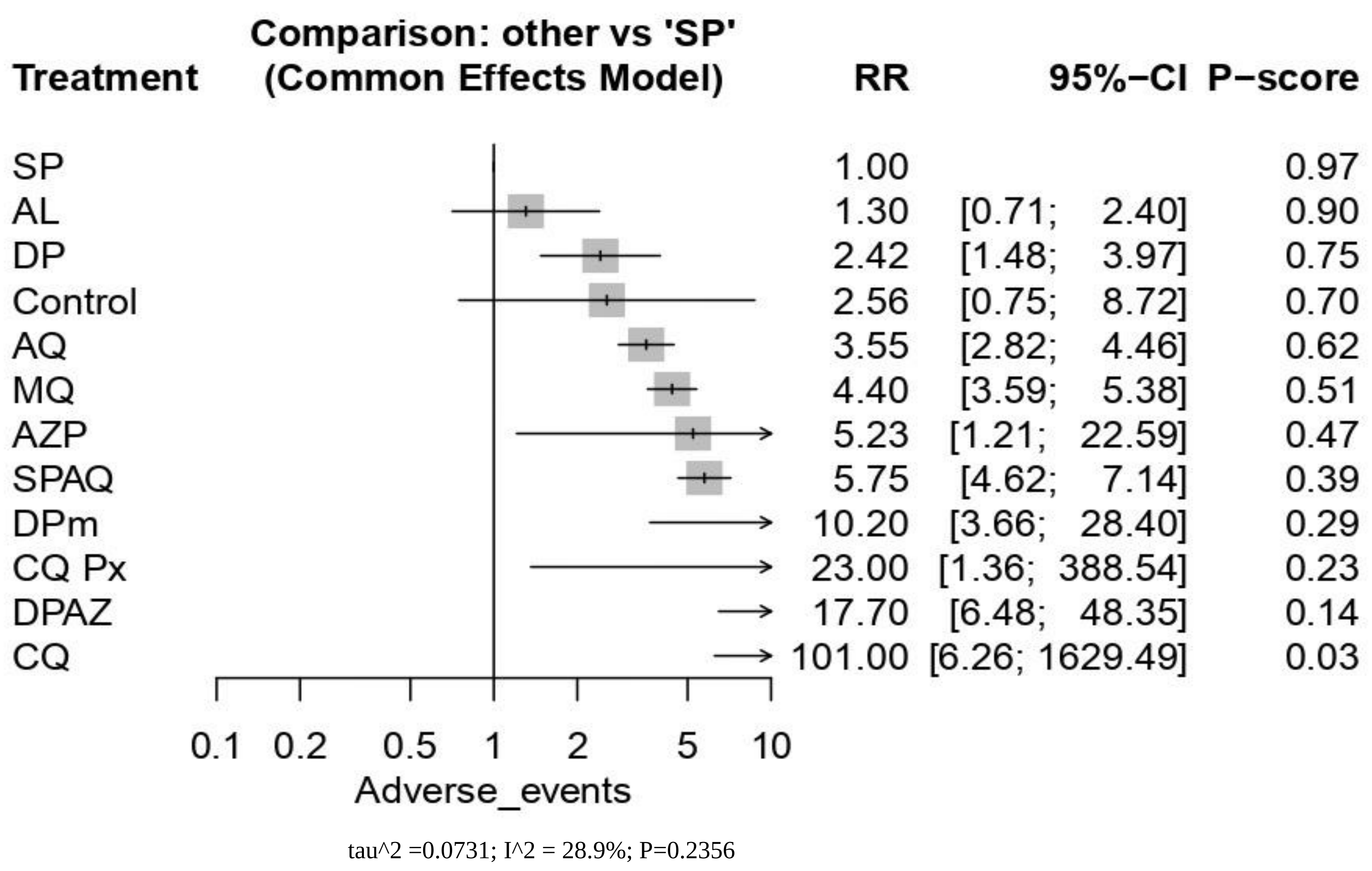

C

**SP**											
0.77 [0.42; 1.41]	**AL**										
0.41 [0.25; 0.68]	0.54 [0.29; 1.01]	**DP**									
0.39 [0.11; 1.33]	0.51 [0.13; 1.95]	0.95 [0.26; 3.45]	**Control**								
**0.28 [0.22; 0.35]**	**0.37 [0.19; 0.71]**	0.68 [0.40; 1.18]	0.72 [0.21; 2.51]	**AQ**							
**0.23 [0.19; 0.28]**	**0.30 [0.17; 0.53]**	0.55 [0.35; 0.87]	0.58 [0.17; 1.95]	0.81 [0.59; 1.09]	**MQ**						
**0.19 [0.04; 0.83]**	**0.25 [0.05; 1.22]**	0.46 [0.10; 2.17]	0.49 [0.07; 3.30]	0.68 [0.15; 2.98]	0.84 [0.19; 3.68]	**AZP**					
**0.17 [0.14; 0.22]**	**0.23 [0.12; 0.43]**	**0.42 [0.25; 0.72]**	0.44 [0.13; 1.55]	0.62 [0.55; 0.69]	0.76 [0.57; 1.03]	0.91 [0.21; 3.99]	**SPAQ**				
**0.10 [0.04; 0.27]**	**0.13 [0.04; 0.42]**	**0.24 [0.08; 0.74]**	0.25 [0.05; 1.24]	0.35 [0.12; 0.99]	0.43 [0.15; 1.22]	0.51 [0.09; 3.06]	0.56 [0.20; 1.61]	**DPm**			
**0.04 [0.00; 0.73]**	**0.06 [0.00; 1.02]**	**0.11 [0.01; 1.86]**	0.11 [0.01; 2.42]	0.15 [0.01; 2.63]	0.19 [0.01; 3.25]	0.23 [0.01; 5.48]	0.25 [0.01; 4.26]	0.44 [0.02; 8.97]	**CQ Px**		
**0.06 [0.02; 0.15]**	**0.07 [0.02; 0.24]**	**0.14 [0.04; 0.42]**	**0.14 [0.03; 0.71]**	**0.20 [0.07; 0.56]**	**0.25 [0.09; 0.69]**	0.30 [0.05; 1.74]	**0.32 [0.12; 0.91]**	**0.58 [0.39; 0.84]**	1.30 [0.06; 26.10]	**DPAZ**	
**0.01 [0.00; 0.16]**	**0.01 [0.00; 0.22]**	**0.02 [0.00; 0.40]**	**0.03 [0.00; 0.53]**	**0.04 [0.00; 0.57]**	**0.04 [0.00; 0.71]**	0.05 [0.00; 1.20]	**0.06 [0.00; 0.93]**	0.10 [0.01; 1.96]	**0.23 [0.12; 0.42]**	0.18 [0.01; 3.37]	**CQ**
