# Supplementary material for: Evaluating the Safety and Efficacy of Malaria Preventive Measures in Pregnant Women with a Focus on HIV Status: A Systematic Review and Network Meta-Analysis
Source: J Clin Med. 2025 May 13;14(10):3396. doi: 10.3390/jcm14103396 (PMC12112236; doi:10.3390/jcm14103396)
Supplement: Supplementary file 1 [file jcm-14-03396-s001.zip › Table S1.pdf]

Table S1: Detailed search strategy for the retrieved databases.

Databases	Search Strategy	Results
**Pubmed**	(((Malaria OR Plasmodium OR Parasitemia OR Paludism OR Falciparum OR VIVAX OR Mosquitos OR Vector OR Culicidae OR culex OR Avian) AND (Nets OR Net OR ITN OR "indoor residual spraying" OR IRS OR Vector OR Control OR RTS,S OR Quinine OR Chloroquine OR Hydroxychloroquine OR Amodiaquine OR Pyrimethamine OR Proguanil OR Sulfonamides OR Mefloquine OR Atovaquone OR Primaquine OR Artemisinin OR Halofantrine OR Lumefantrine OR Doxycycline OR Clindamycin OR Mefloquine OR Lariam OR Pyrimethamine OR Daraprim OR Artemether OR Artesunate OR Dihydroartemisinin OR Arteether OR ACT OR Non-ACT OR Prevention OR Control OR Preventive OR Insecticide* OR Insecticidal OR Anti-malarials OR Antimalaria OR Spray OR Spraying OR Bednets)) AND (Pregnancy OR Pregnant OR Pregnancies))	9045
**Scopus**	(((Malaria OR Plasmodium OR Parasitemia OR Paludism OR Falciparum OR VIVAX OR Mosquitos OR Vector OR Culicidae OR culex OR Avian) AND (Nets OR Net OR ITN OR "indoor residual spraying" OR IRS OR Vector OR Control OR RTS,S OR Quinine OR Chloroquine OR Hydroxychloroquine OR Amodiaquine OR Pyrimethamine OR Proguanil OR Sulfonamides OR Mefloquine OR Atovaquone OR Primaquine OR Artemisinin OR Halofantrine OR Lumefantrine OR Doxycycline OR Clindamycin OR Mefloquine OR Lariam OR Pyrimethamine OR Daraprim OR Artemether OR Artesunate OR Dihydroartemisinin OR Arteether OR ACT OR Non-ACT OR Prevention	5018

	OR Control OR Preventive OR Insecticide* OR Insecticidal OR Anti-malarials OR Antimalaria OR Spray OR Spraying OR Bednets)) AND (Pregnancy OR Pregnant OR Pregnancies))	
**Web of science**	(((Malaria OR Plasmodium OR Parasitemia OR Paludism OR Falciparum OR VIVAX OR Mosquitos OR Vector OR Culicidae OR culex OR Avian) AND (Nets OR Net OR ITN OR “indoor residual spraying” OR IRS OR Vector OR Control OR RTS,S OR Quinine OR Chloroquine OR Hydroxychloroquine OR Amodiaquine OR Pyrimethamine OR Proguanil OR Sulfonamides OR Mefloquine OR Atovaquone OR Primaquine OR Artemisinin OR Halofantrine OR Lumefantrine OR Doxycycline OR Clindamycin OR Mefloquine OR Lariam OR Pyrimethamine OR Daraprim OR Artemether OR Artesunate OR Dihydroartemisinin OR Arteether OR ACT OR Non-ACT OR Prevention OR Control OR Preventive OR Insecticide* OR Insecticidal OR Anti-malarials OR Antimalaria OR Spray OR Spraying OR Bednets)) AND (Pregnancy OR Pregnant OR Pregnancies))	5745
**Cochrane library**	(((Malaria OR Plasmodium OR Parasitemia OR Paludism OR Falciparum OR VIVAX OR Mosquitos OR Vector OR Culicidae OR culex OR Avian) AND (Nets OR Net OR ITN OR “indoor residual spraying” OR IRS OR Vector OR Control OR RTS,S OR Quinine OR Chloroquine OR Hydroxychloroquine OR Amodiaquine OR Pyrimethamine OR Proguanil OR Sulfonamides OR Mefloquine OR Atovaquone OR Primaquine OR Artemisinin OR Halofantrine OR Lumefantrine OR Doxycycline OR Clindamycin OR Mefloquine OR Lariam OR Pyrimethamine OR Daraprim OR Artemether OR Artesunate OR Dihydroartemisinin OR Arteether OR ACT OR Non-ACT OR Prevention OR Control OR Preventive OR Insecticide* OR Insecticidal OR Anti-malarials OR Antimalaria OR Spray OR Spraying OR Bednets)) AND (Pregnancy OR Pregnant OR Pregnancies))	1086
