# Supplementary material for: Evaluating the Safety and Efficacy of Malaria Preventive Measures in Pregnant Women with a Focus on HIV Status: A Systematic Review and Network Meta-Analysis
Source: J Clin Med. 2025 May 13;14(10):3396. doi: 10.3390/jcm14103396 (PMC12112236; doi:10.3390/jcm14103396)
Supplement: Supplementary file 1 [file jcm-14-03396-s001.zip › Table S2.pdf]

ID	Table S2: Quality assessment of Quasi-experimental studies												Quality rating: Good (9-11) or Fair (6-8.5) or Poor (5.5-0)
	1. Was an attempt made to blind those measuring the intervention?	2. Have the characteristics of participants (Likely clininicians) lost to follow-up been described?	3. Do the analysis adjust for different lengths of follow-up of participants?	4. Were the statistical tests used to assess the main outcomes appropriate?	5. Was Compliance with the intervention reliable?	6. Were the main outcome measures clearly defined, valid, reliable, and implemented consistently across all study participants?	7. Was the length of follow-up adequate?	8. Were participants in different intervention groups recruited from the same population?	9. Were participants in different intervention groups recruited from the same period of time?	10. Was there adequate adjustment for confounding in the analysis from which the main findings were drawn?	11. Were loses of participants to follow-up taken into account?	Total scores: Yes = 1 // No = 0.5 // NR & NA & CD = 0	
	Yes / No / Not reported (NR) or cannot determine (CD) or not applicable (NA)	Yes / No / Not reported (NR) or cannot determine (CD) or not applicable (NA)	Yes / No / Not reported (NR) or cannot determine (CD) or not applicable (NA)	Yes / No / Not reported (NR) or cannot determine (CD) or not applicable (NA)	Yes / No / Not reported (NR) or cannot determine (CD) or not applicable (NA)	Yes / No / Not reported (NR) or cannot determine (CD) or not applicable (NA)	Yes / No / Not reported (NR) or cannot determine (CD) or not applicable (NA)	Yes / No / Not reported (NR) or cannot determine (CD) or not applicable (NA)	Yes / No / Not reported (NR) or cannot determine (CD) or not applicable (NA)	Yes / No / Not reported (NR) or cannot determine (CD) or not applicable (NA)	Yes / No / Not reported (NR) or cannot determine (CD) or not applicable (NA)		
**Kumar et.al 2020**	NR	NR	NR	Yes	Yes	Yes	Yes	Yes	Yes	Yes	NR	**7**	**Fair**
**Roh et.al 2022**	NR	Yes	Yes	Yes	Yes	NR	Yes	Yes	Yes	Yes	Yes	**9**	**Good**
