# Supplementary figures and images for: Evaluating the Safety and Efficacy of Malaria Preventive Measures in Pregnant Women with a Focus on HIV Status: A Systematic Review and Network Meta-Analysis
Source: J Clin Med. 2025 May 13;14(10):3396. doi: 10.3390/jcm14103396 (PMC12112236; doi:10.3390/jcm14103396)

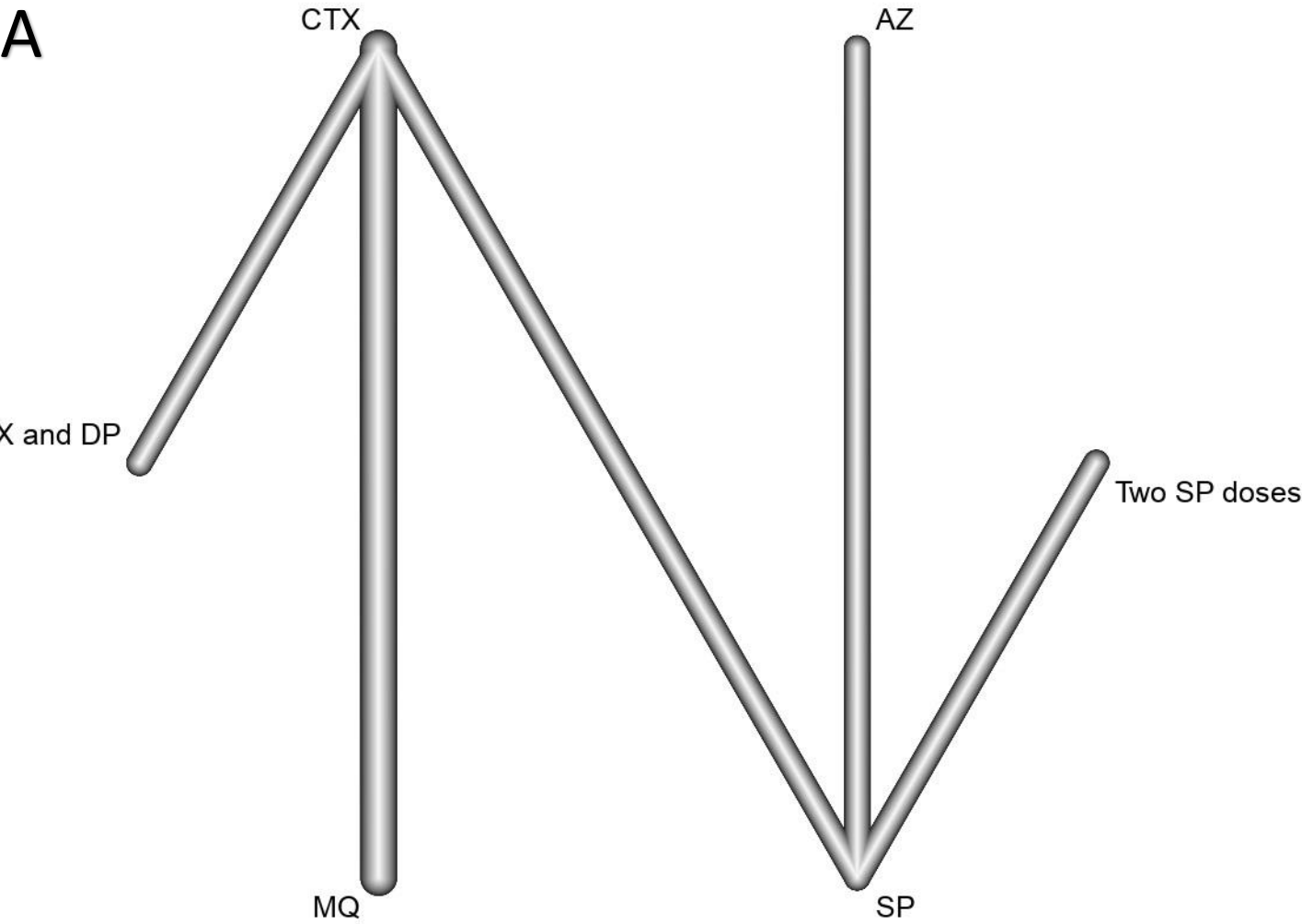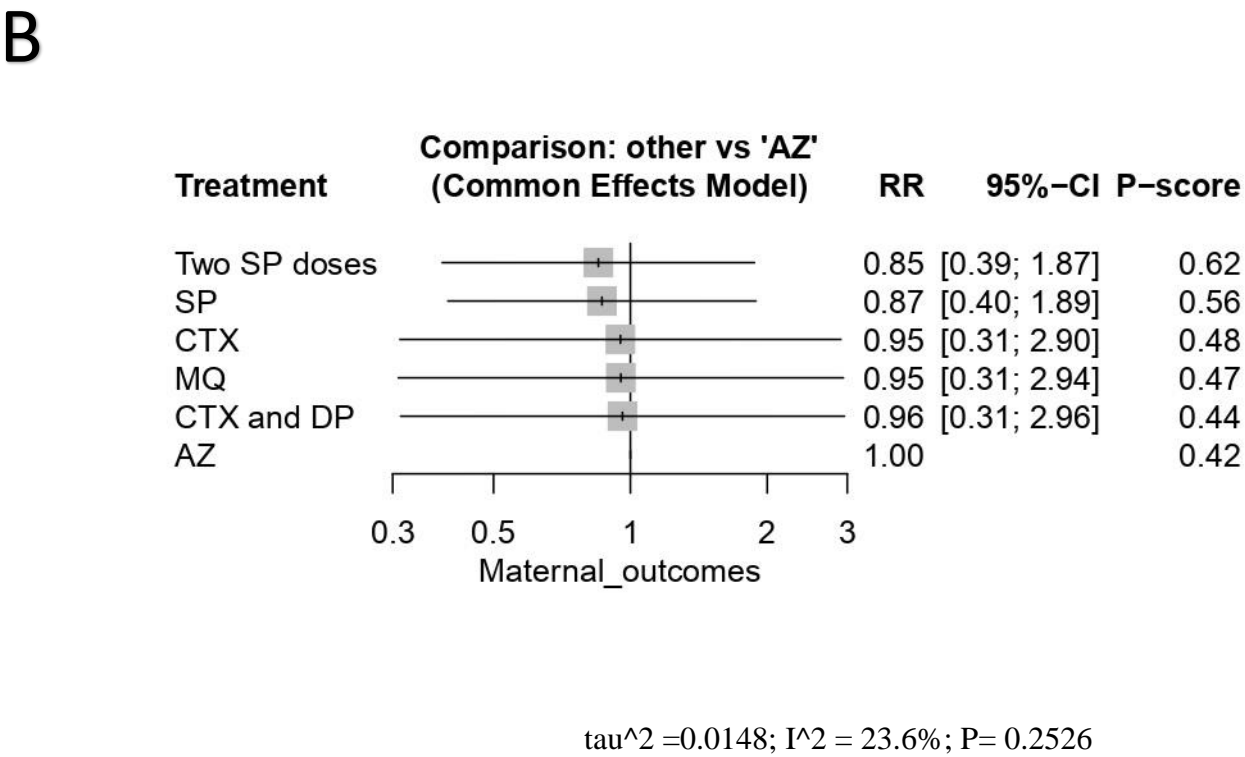

**C**

|                   |                   |                   |                   |                   |    |
|-------------------|-------------------|-------------------|-------------------|-------------------|----|
| Two SP doses      |                   |                   |                   |                   |    |
| 0.98 [0.86; 1.12] | SP                |                   |                   |                   |    |
| 0.89 [0.40; 2.01] | 0.91 [0.41; 2.03] | CTX               |                   |                   |    |
| 0.89 [0.39; 2.03] | 0.91 [0.40; 2.05] | 1.00 [0.86; 1.16] | MQ                |                   |    |
| 0.88 [0.39; 2.01] | 0.90 [0.40; 2.02] | 0.99 [0.87; 1.12] | 0.99 [0.81; 1.21] | CTX and DP        |    |
| 0.85 [0.39; 1.87] | 0.87 [0.40; 1.89] | 0.95 [0.31; 2.90] | 0.95 [0.31; 2.94] | 0.96 [0.31; 2.96] | AZ |

Supplement: Supplementary file 1 [file jcm-14-03396-s001.zip › Figure S1.pdf]

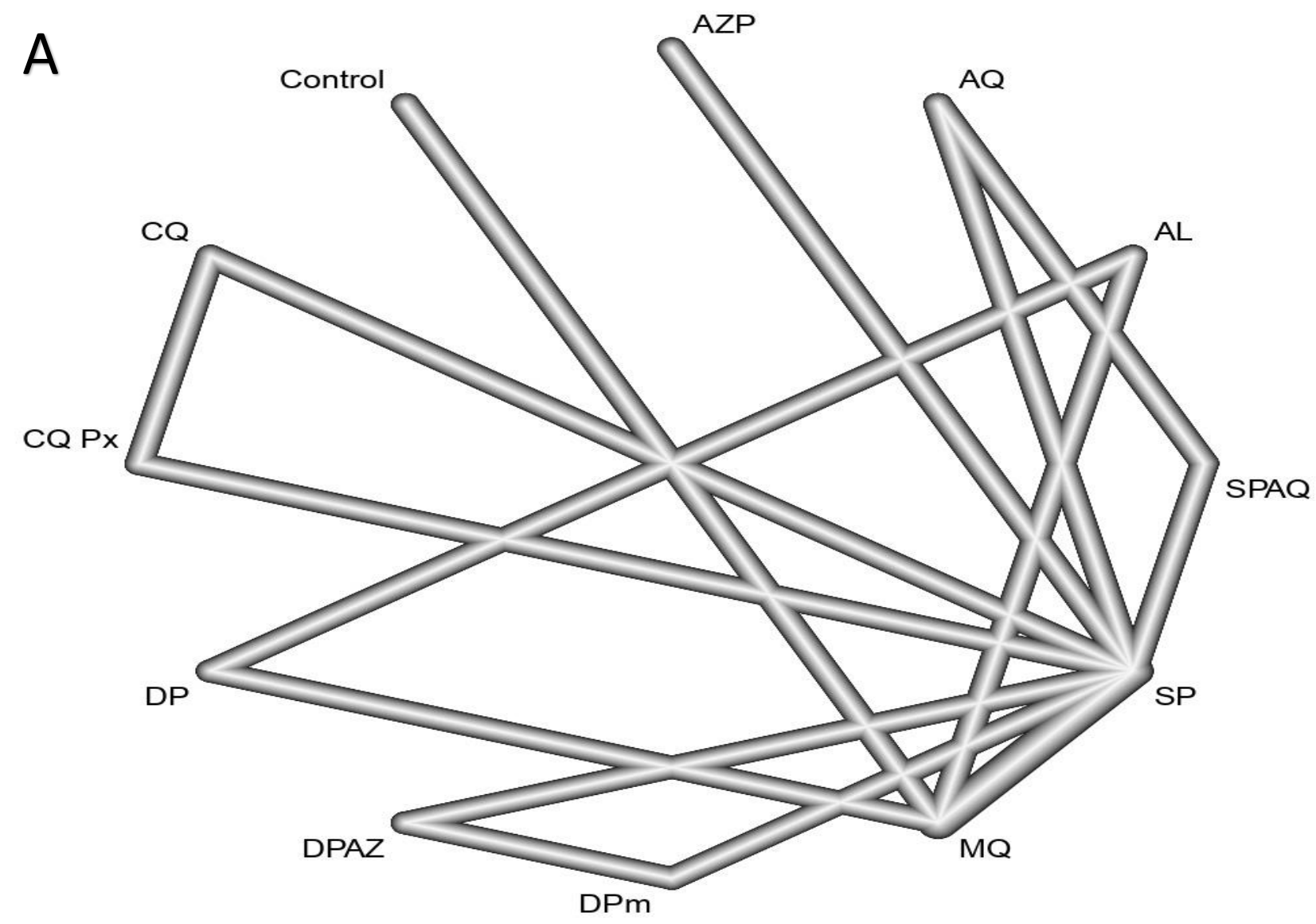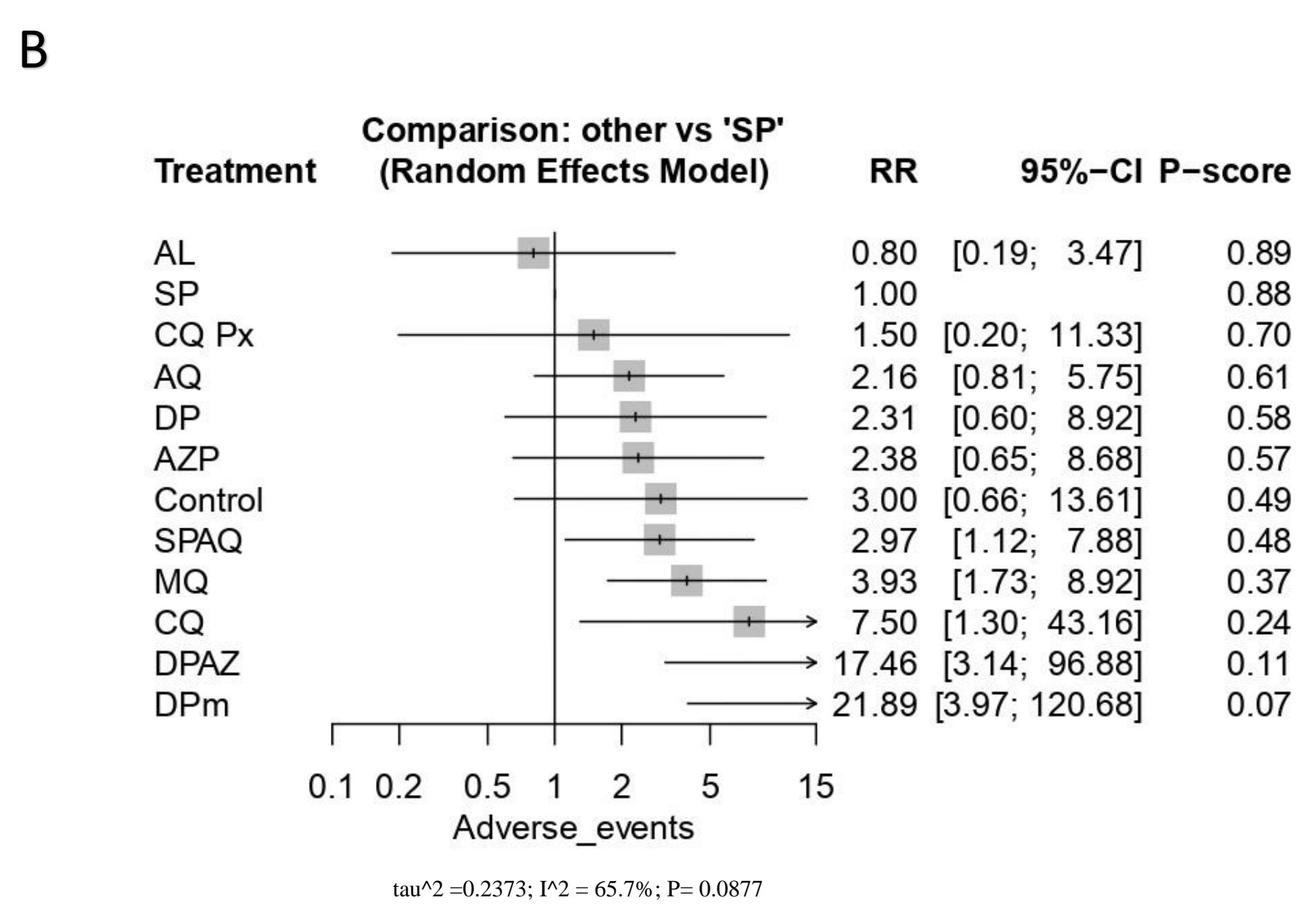

**C**

|                   |                   |                   |                   |                   |                   |                   |                   |                   |                   |                   |     |  |
|-------------------|-------------------|-------------------|-------------------|-------------------|-------------------|-------------------|-------------------|-------------------|-------------------|-------------------|-----|--|
| AL                |                   |                   |                   |                   |                   |                   |                   |                   |                   |                   |     |  |
| 0.80 [0.19; 3.47] | SP                |                   |                   |                   |                   |                   |                   |                   |                   |                   |     |  |
| 0.54 [0.04; 6.49] | 0.67 [0.09; 5.03] | CQ Px             |                   |                   |                   |                   |                   |                   |                   |                   |     |  |
| 0.37 [0.06; 2.16] | 0.46 [0.17; 1.23] | 0.69 [0.07; 6.55] | AQ                |                   |                   |                   |                   |                   |                   |                   |     |  |
| 0.35 [0.10; 1.20] | 0.43 [0.11; 1.67] | 0.65 [0.06; 7.38] | 0.94 [0.18; 4.96] | DP                |                   |                   |                   |                   |                   |                   |     |  |
| 0.34 [0.05; 2.38] | 0.42 [0.12; 1.54] | 0.63 [0.06; 6.96] | 0.91 [0.18; 4.62] | 0.97 [0.15; 6.32] | AZP               |                   |                   |                   |                   |                   |     |  |
| 0.27 [0.05; 1.55] | 0.33 [0.07; 1.51] | 0.50 [0.04; 6.24] | 0.72 [0.12; 4.36] | 0.77 [0.15; 4.06] | 0.79 [0.11; 5.80] | Control           |                   |                   |                   |                   |     |  |
| 0.27 [0.05; 1.57] | 0.34 [0.13; 0.89] | 0.51 [0.05; 4.77] | 0.73 [0.28; 1.91] | 0.78 [0.15; 4.12] | 0.80 [0.16; 4.06] | 1.01 [0.17; 6.11] | SPAQ              |                   |                   |                   |     |  |
| 0.20 [0.06; 0.69] | 0.25 [0.11; 0.58] | 0.38 [0.04; 3.38] | 0.55 [0.15; 1.97] | 0.59 [0.20; 1.72] | 0.60 [0.13; 2.80] | 0.76 [0.21; 2.72] | 0.75 [0.21; 2.70] | MQ                |                   |                   |     |  |
| 0.11 [0.01; 1.05] | 0.13 [0.02; 0.77] | 0.20 [0.04; 0.95] | 0.29 [0.04; 2.14] | 0.31 [0.03; 2.81] | 0.32 [0.04; 2.80] | 0.40 [0.04; 4.04] | 0.40 [0.05; 2.94] | 0.52 [0.08; 3.62] | CQ                |                   |     |  |
| 0.05 [0.00; 0.44] | 0.06 [0.01; 0.32] | 0.09 [0.01; 1.22] | 0.12 [0.02; 0.89] | 0.13 [0.01; 1.17] | 0.14 [0.02; 1.17] | 0.17 [0.02; 1.69] | 0.17 [0.02; 1.22] | 0.23 [0.03; 1.51] | 0.43 [0.04; 4.98] | DPAZ              |     |  |
| 0.04 [0.00; 0.35] | 0.05 [0.01; 0.25] | 0.07 [0.00; 0.97] | 0.10 [0.01; 0.71] | 0.11 [0.01; 0.93] | 0.11 [0.01; 0.93] | 0.14 [0.01; 1.34] | 0.14 [0.02; 0.97] | 0.18 [0.03; 1.19] | 0.34 [0.03; 3.95] | 0.80 [0.28; 2.28] | DPm |  |

Supplement: Supplementary file 1 [file jcm-14-03396-s001.zip › Figure S10.pdf]

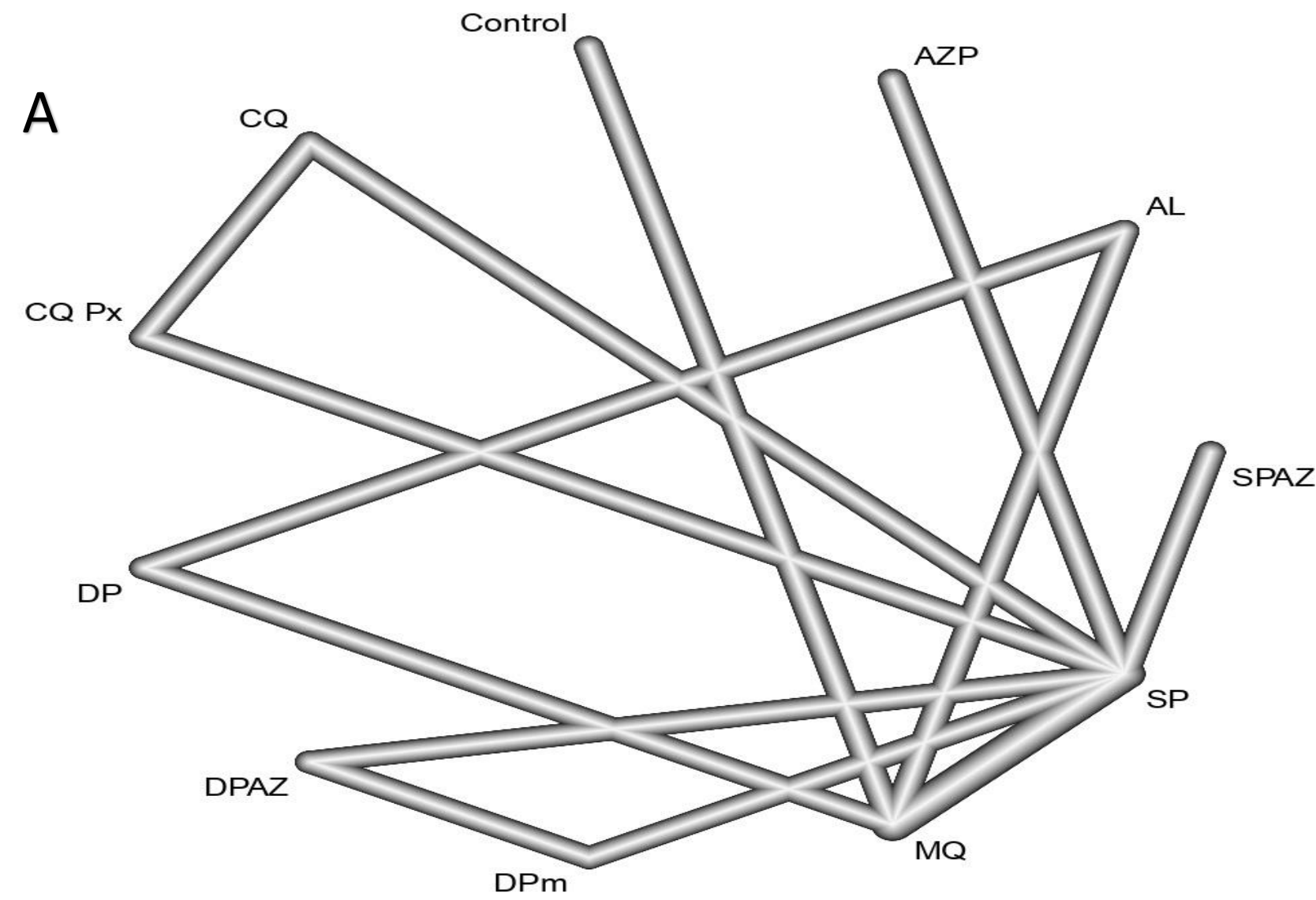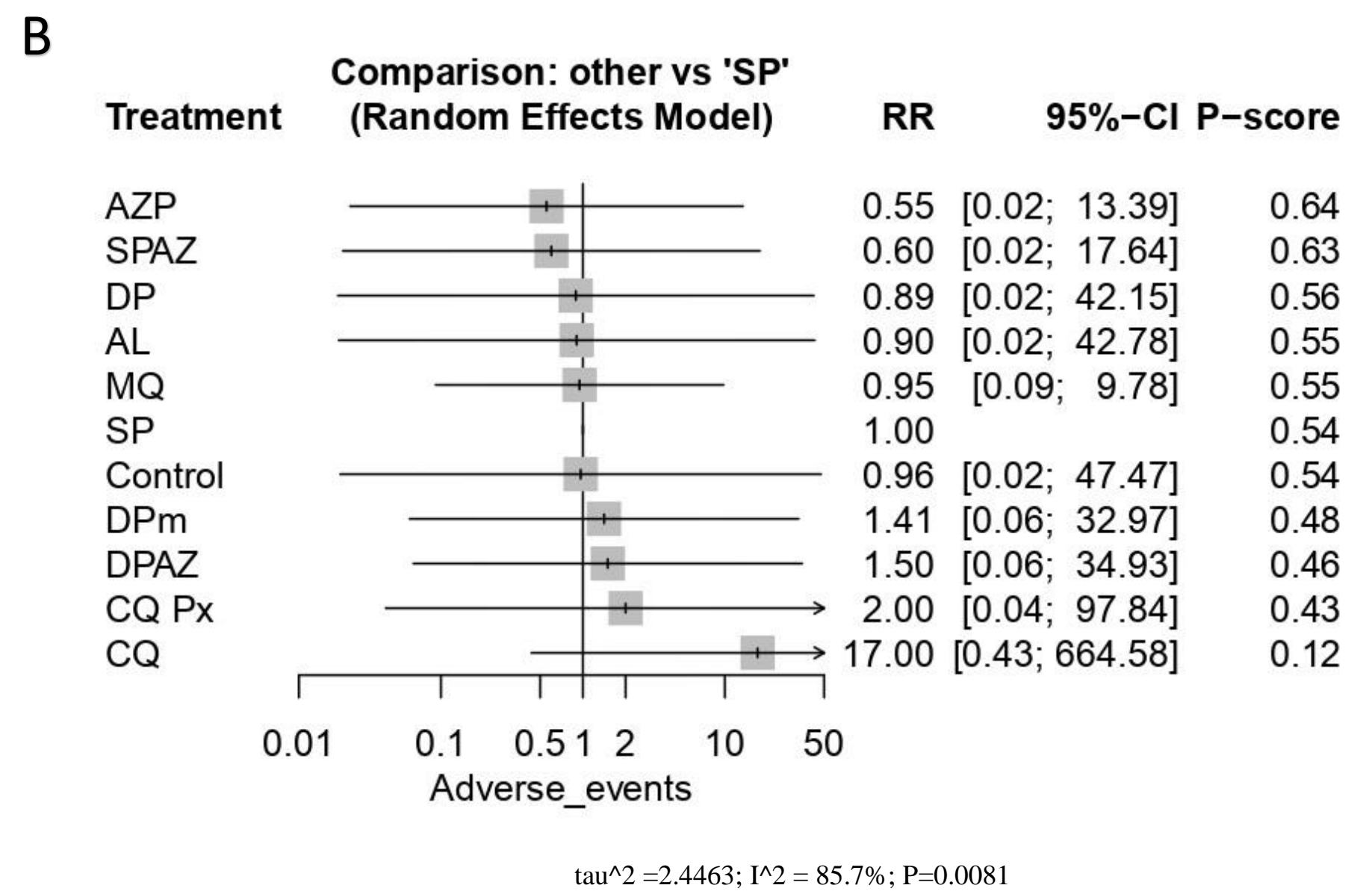

**C**

| AZP                |                     |                     |                     |                    |                    |                     |                     |                     |                   |    |
|--------------------|---------------------|---------------------|---------------------|--------------------|--------------------|---------------------|---------------------|---------------------|-------------------|----|
| 0.92 [0.01; 96.11] | SPAZ                |                     |                     |                    |                    |                     |                     |                     |                   |    |
| 0.62 [0.00; 92.52] | 0.67 [0.00; 113.72] | DP                  |                     |                    |                    |                     |                     |                     |                   |    |
| 0.61 [0.00; 91.15] | 0.66 [0.00; 112.04] | 0.99 [0.05; 21.24]  | AL                  |                    |                    |                     |                     |                     |                   |    |
| 0.59 [0.01; 30.35] | 0.63 [0.01; 38.54]  | 0.94 [0.04; 20.27]  | 0.95 [0.04; 20.57]  | MQ                 |                    |                     |                     |                     |                   |    |
| 0.55 [0.02; 13.39] | 0.60 [0.02; 17.64]  | 0.89 [0.02; 42.15]  | 0.90 [0.02; 42.78]  | 0.95 [0.09; 9.78]  | SP                 |                     |                     |                     |                   |    |
| 0.58 [0.00; 88.16] | 0.62 [0.00; 108.28] | 0.92 [0.01; 73.60]  | 0.94 [0.01; 74.69]  | 0.98 [0.04; 22.26] | 1.04 [0.02; 51.07] | Control             |                     |                     |                   |    |
| 0.39 [0.00; 34.74] | 0.43 [0.00; 43.32]  | 0.63 [0.00; 92.08]  | 0.64 [0.00; 93.45]  | 0.67 [0.01; 33.96] | 0.71 [0.03; 16.60] | 0.68 [0.00; 102.78] | DPm                 |                     |                   |    |
| 0.37 [0.00; 32.68] | 0.40 [0.00; 40.76]  | 0.60 [0.00; 86.63]  | 0.60 [0.00; 87.92]  | 0.63 [0.01; 31.94] | 0.67 [0.03; 15.61] | 0.64 [0.00; 96.70]  | 0.94 [0.04; 21.67]  | DPAZ                |                   |    |
| 0.28 [0.00; 42.29] | 0.30 [0.00; 51.94]  | 0.45 [0.00; 106.63] | 0.45 [0.00; 108.22] | 0.47 [0.01; 44.23] | 0.50 [0.01; 24.46] | 0.48 [0.00; 118.69] | 0.70 [0.00; 105.35] | 0.75 [0.01; 111.69] | CQ Px             |    |
| 0.03 [0.00; 4.19]  | 0.04 [0.00; 5.17]   | 0.05 [0.00; 10.72]  | 0.05 [0.00; 10.88]  | 0.06 [0.00; 4.30]  | 0.06 [0.00; 2.30]  | 0.06 [0.00; 11.95]  | 0.08 [0.00; 10.43]  | 0.09 [0.00; 11.06]  | 0.12 [0.00; 3.50] | CQ |

Supplement: Supplementary file 1 [file jcm-14-03396-s001.zip › Figure S12.pdf]

A

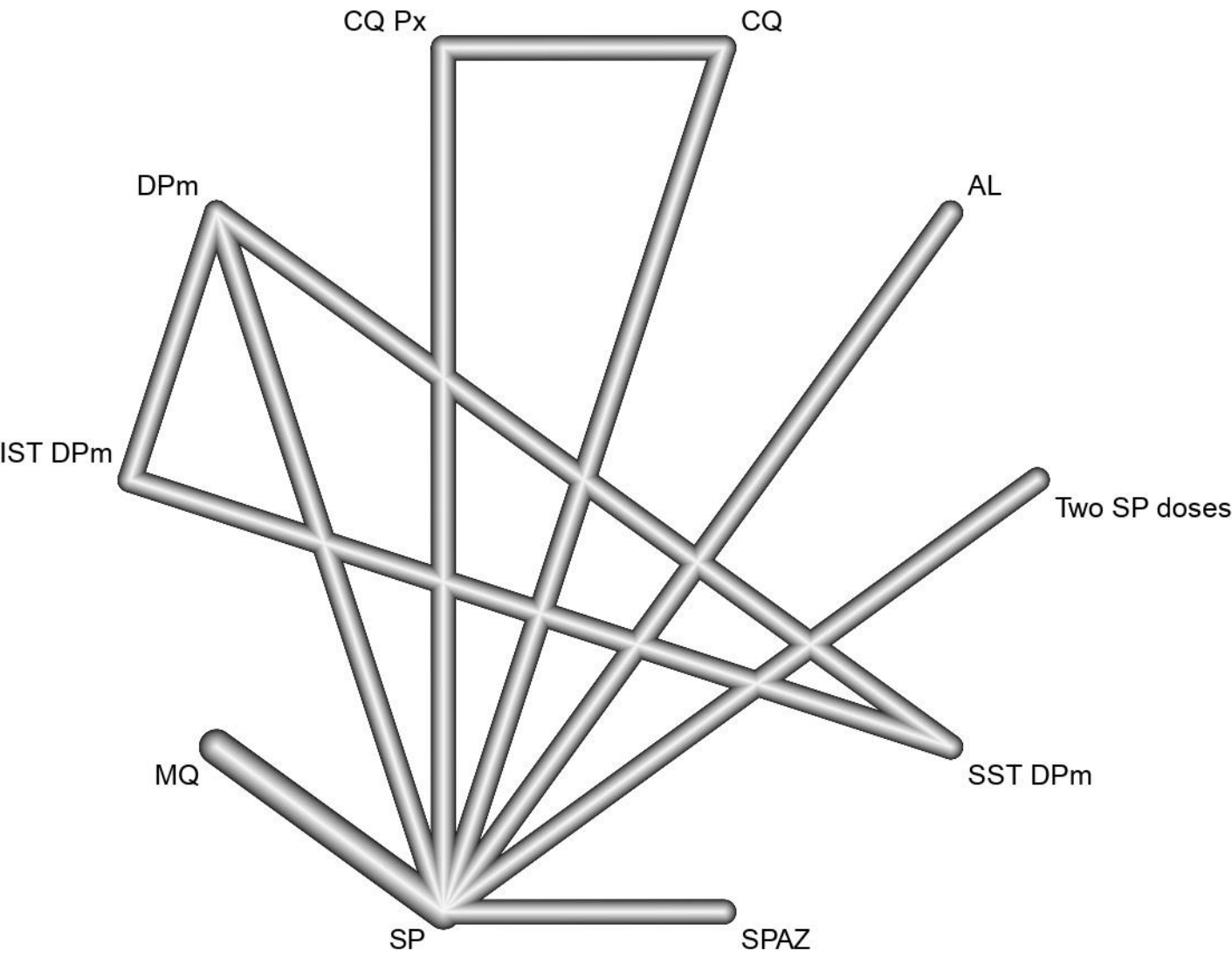

B

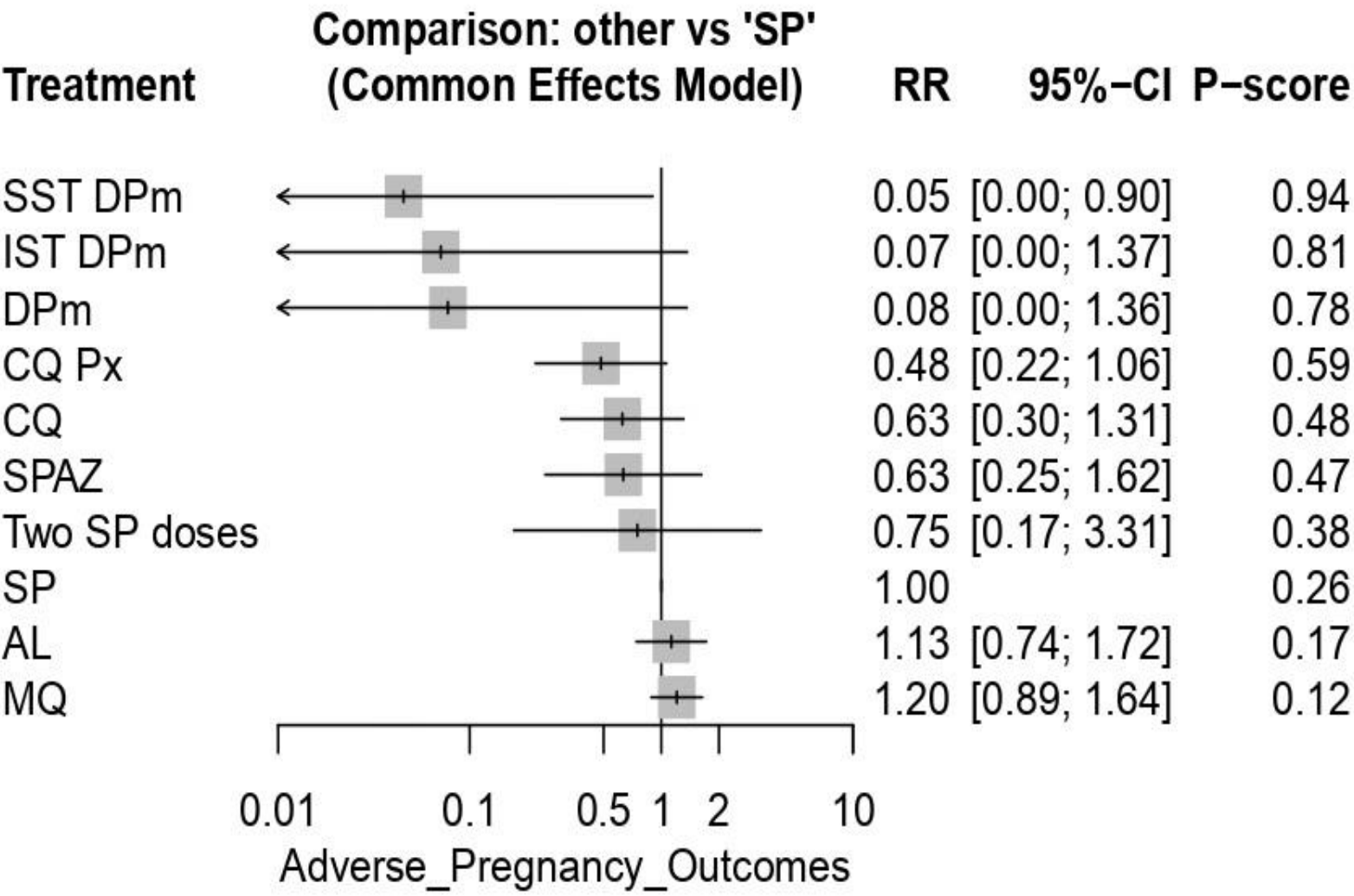

tau^2 =0; I^2 = 0%; P=0.5496

C

|                   |                   |                   |                   |                   |                   |                   |                   |                   |    |  |
|-------------------|-------------------|-------------------|-------------------|-------------------|-------------------|-------------------|-------------------|-------------------|----|--|
| SST DPm           |                   |                   |                   |                   |                   |                   |                   |                   |    |  |
| 0.64 [0.28; 1.44] | IST DPm           |                   |                   |                   |                   |                   |                   |                   |    |  |
| 0.59 [0.25; 1.36] | 0.92 [0.45; 1.89] | DPm               |                   |                   |                   |                   |                   |                   |    |  |
| 0.09 [0.00; 2.06] | 0.15 [0.01; 3.13] | 0.16 [0.01; 3.13] | CQ Px             |                   |                   |                   |                   |                   |    |  |
| 0.07 [0.00; 1.57] | 0.11 [0.01; 2.39] | 0.12 [0.01; 2.39] | 0.77 [0.32; 1.84] | CQ                |                   |                   |                   |                   |    |  |
| 0.07 [0.00; 1.64] | 0.11 [0.00; 2.50] | 0.12 [0.01; 2.50] | 0.76 [0.22; 2.60] | 0.99 [0.30; 3.26] | SPAZ              |                   |                   |                   |    |  |
| 0.06 [0.00; 1.70] | 0.09 [0.00; 2.59] | 0.10 [0.00; 2.60] | 0.65 [0.12; 3.47] | 0.84 [0.16; 4.38] | 0.84 [0.15; 4.89] | Two SP doses      |                   |                   |    |  |
| 0.05 [0.00; 0.90] | 0.07 [0.00; 1.37] | 0.08 [0.00; 1.36] | 0.48 [0.22; 1.06] | 0.63 [0.30; 1.31] | 0.63 [0.25; 1.62] | 0.75 [0.17; 3.31] | SP                |                   |    |  |
| 0.04 [0.00; 0.82] | 0.06 [0.00; 1.25] | 0.07 [0.00; 1.24] | 0.43 [0.18; 1.05] | 0.56 [0.24; 1.30] | 0.56 [0.20; 1.57] | 0.66 [0.14; 3.11] | 0.89 [0.58; 1.35] | AL                |    |  |
| 0.04 [0.00; 0.76] | 0.06 [0.00; 1.15] | 0.06 [0.00; 1.15] | 0.40 [0.17; 0.94] | 0.52 [0.23; 1.15] | 0.53 [0.20; 1.41] | 0.62 [0.14; 2.84] | 0.83 [0.61; 1.13] | 0.94 [0.56; 1.57] | MQ |  |

Supplement: Supplementary file 1 [file jcm-14-03396-s001.zip › Figure S13.pdf]

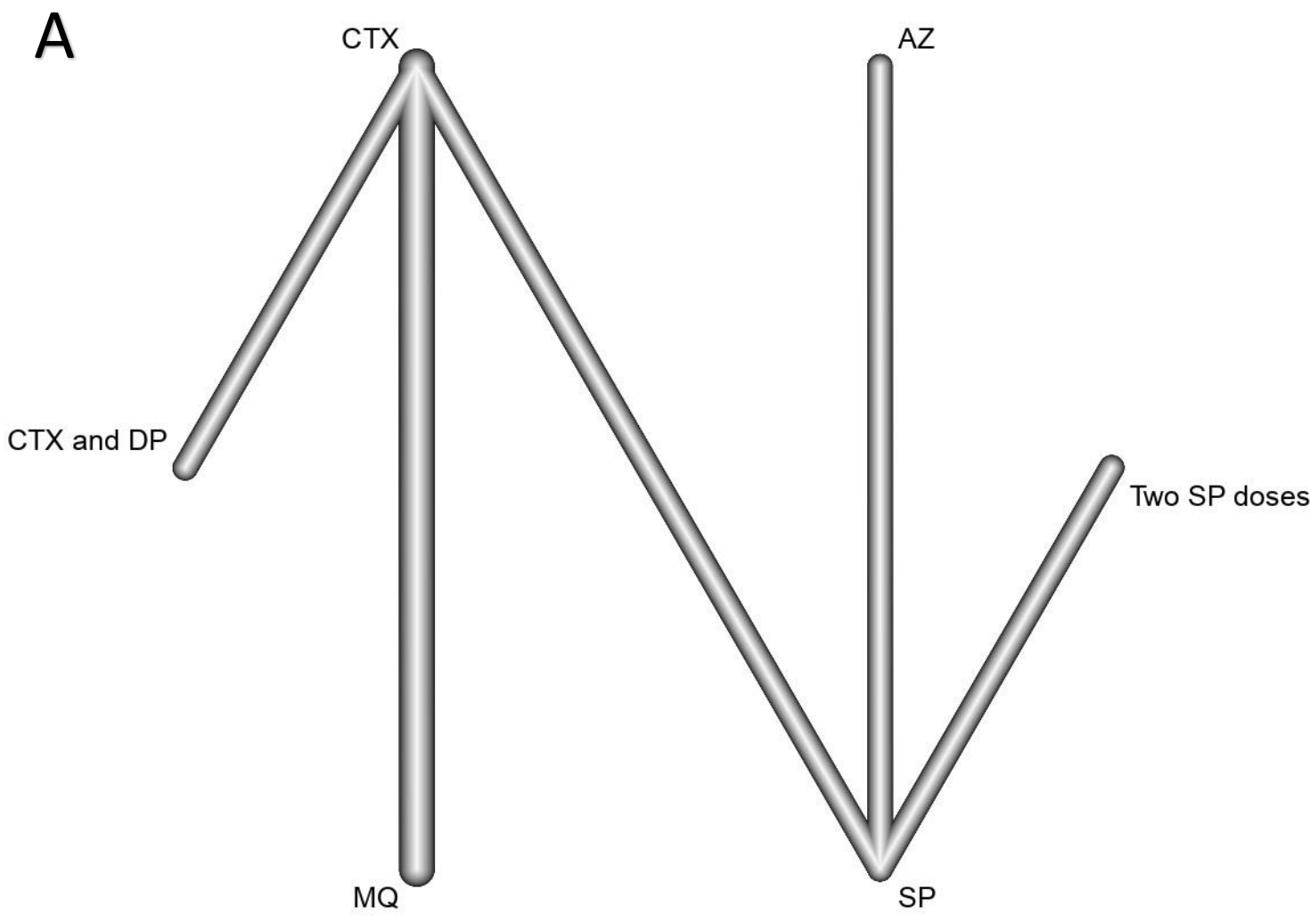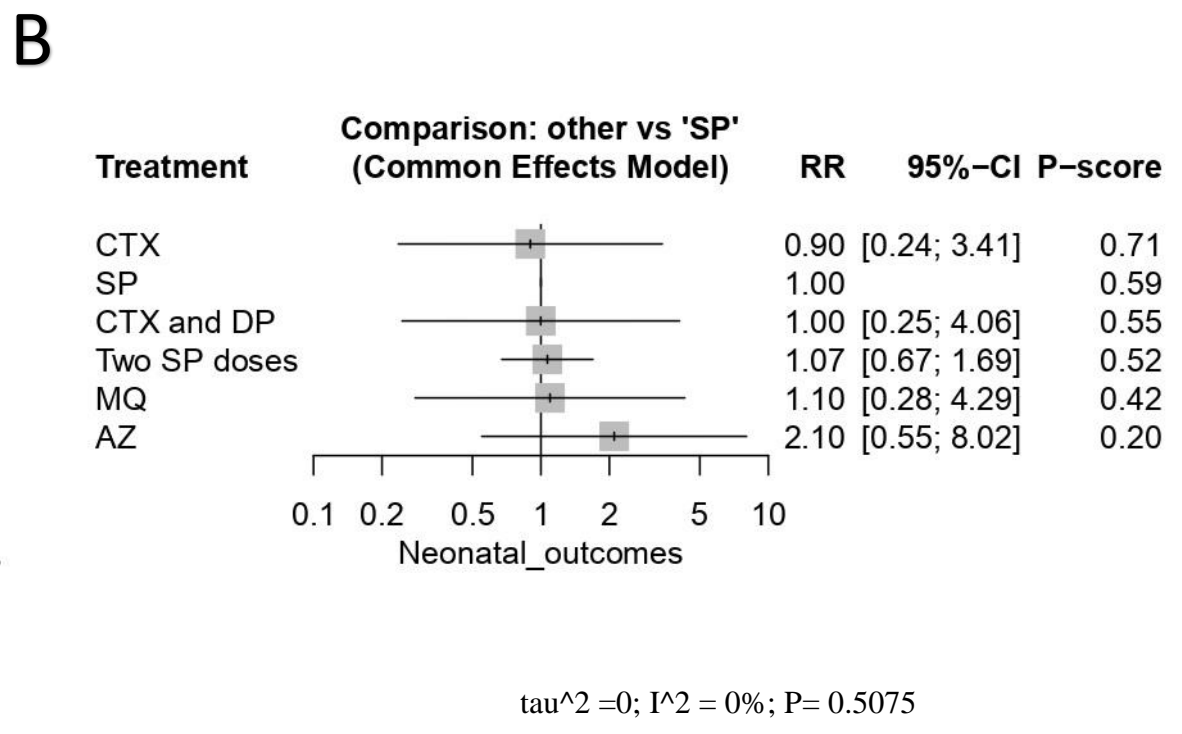

**C**

| CTX               |                   |                   |                   |                   |    |
|-------------------|-------------------|-------------------|-------------------|-------------------|----|
| 0.90 [0.24; 3.41] | SP                |                   |                   |                   |    |
| 0.90 [0.58; 1.39] | 1.00 [0.25; 4.08] | CTX and DP        |                   |                   |    |
| 0.84 [0.20; 3.46] | 0.94 [0.59; 1.48] | 0.94 [0.21; 4.10] | Two SP doses      |                   |    |
| 0.82 [0.62; 1.09] | 0.91 [0.23; 3.57] | 0.91 [0.54; 1.53] | 0.97 [0.23; 4.11] | MQ                |    |
| 0.43 [0.06; 2.84] | 0.48 [0.12; 1.82] | 0.48 [0.07; 3.31] | 0.51 [0.12; 2.10] | 0.52 [0.08; 3.53] | AZ |

Supplement: Supplementary file 1 [file jcm-14-03396-s001.zip › Figure S2.pdf]

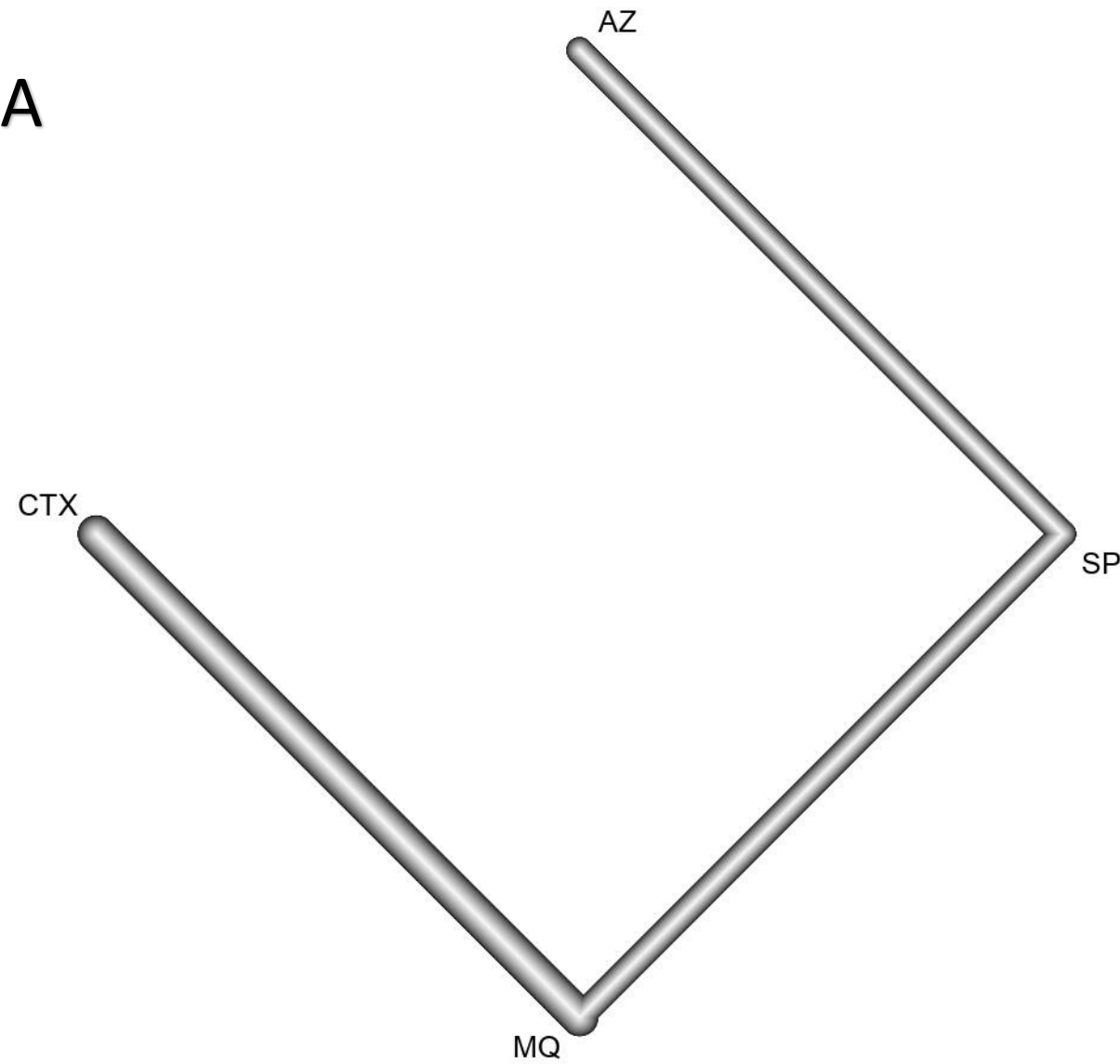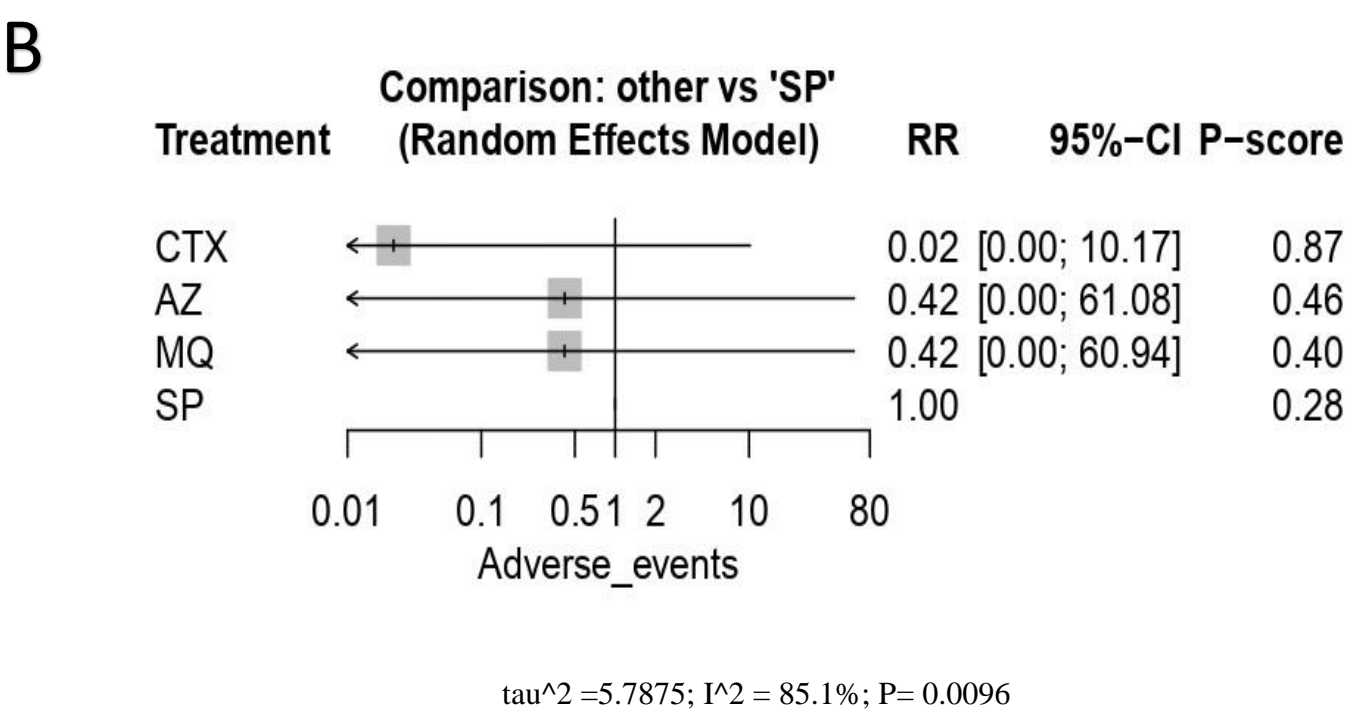

**C**

| CTX                 |                      |                    |    |
|---------------------|----------------------|--------------------|----|
| 0.05 [0.00; 141.82] | AZ                   |                    |    |
| 0.05 [0.00; 1.89]   | 1.00 [0.00; 1147.99] | MQ                 |    |
| 0.02 [0.00; 10.17]  | 0.42 [0.00; 61.08]   | 0.42 [0.00; 60.94] | SP |

Supplement: Supplementary file 1 [file jcm-14-03396-s001.zip › Figure S3.pdf]

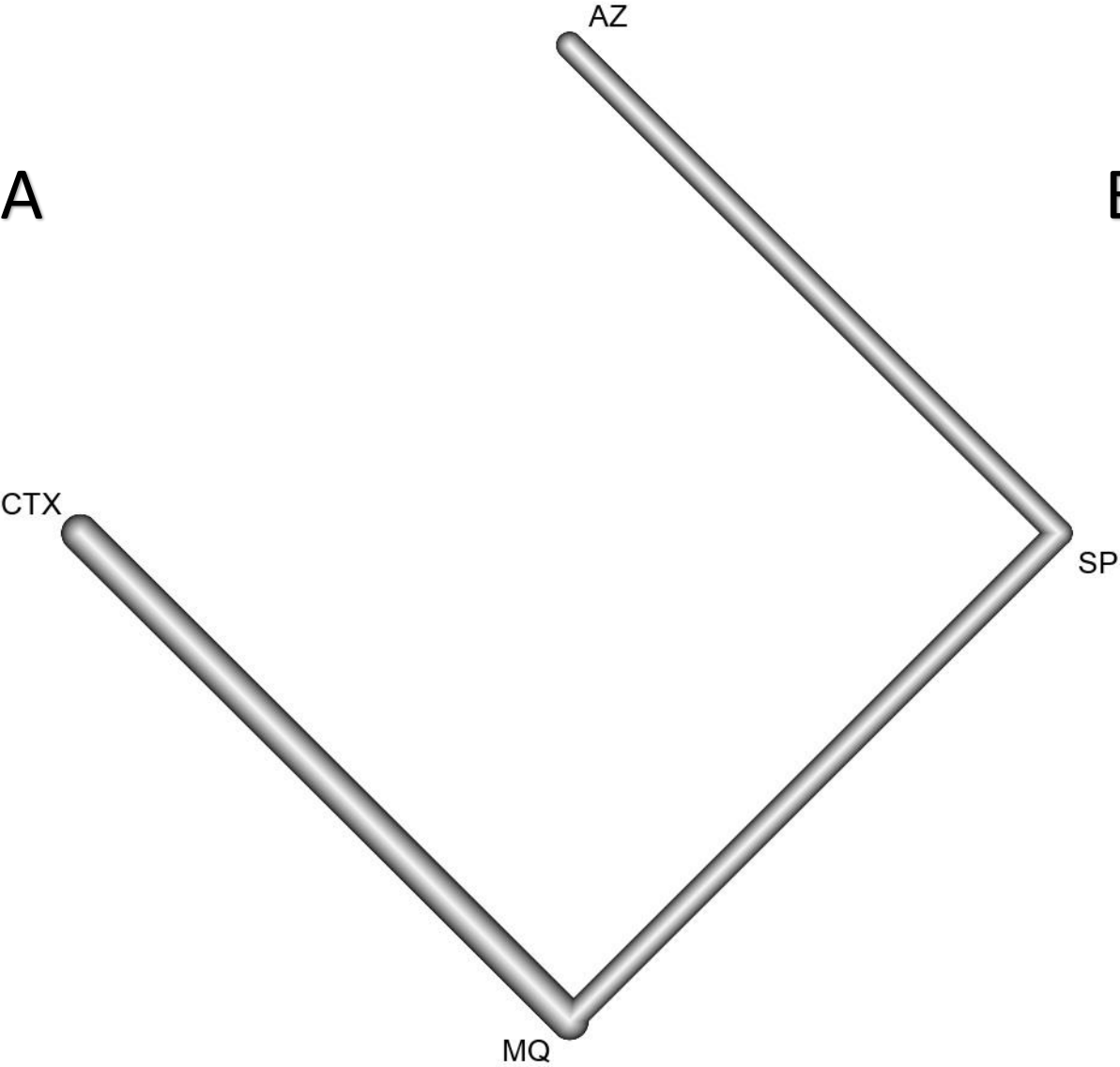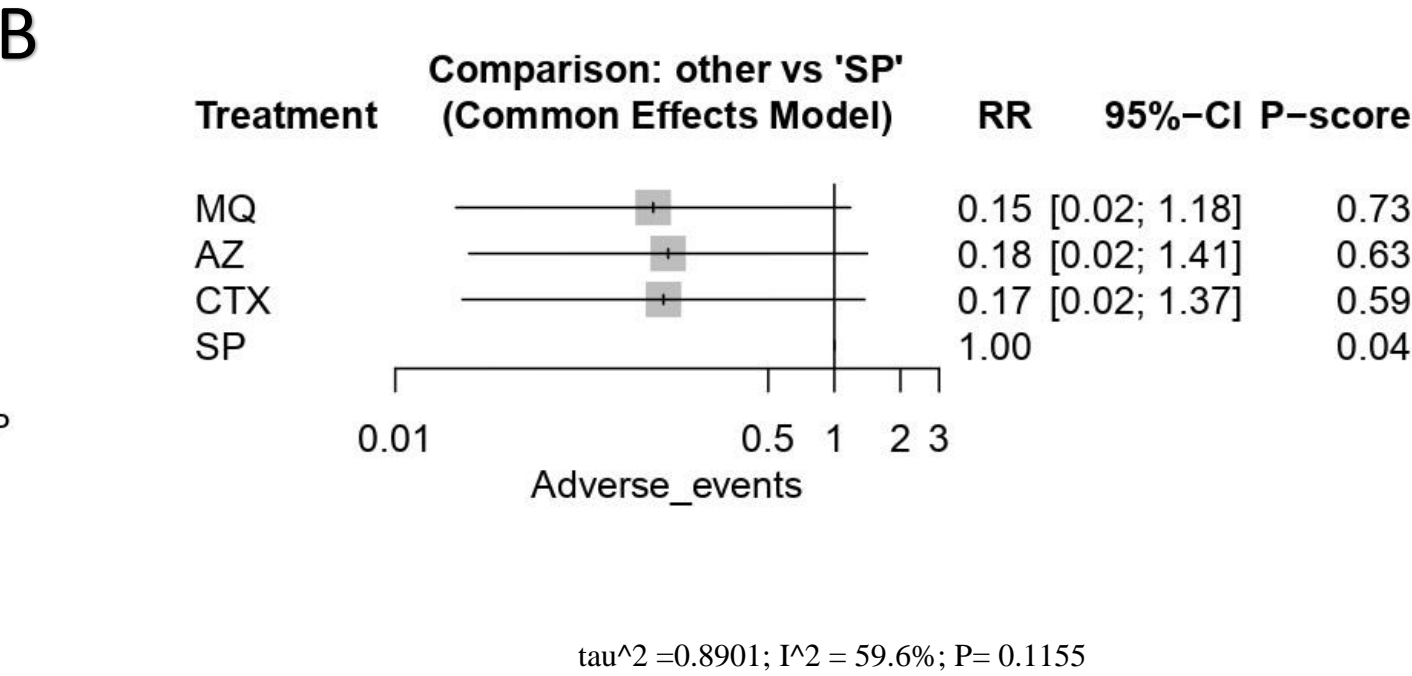

**C**

| MQ                 |                    |                   |    |
|--------------------|--------------------|-------------------|----|
| 0.85 [0.05; 16.13] | AZ                 |                   |    |
| 0.90 [0.59; 1.36]  | 1.05 [0.05; 20.40] | CTX               |    |
| 0.15 [0.02; 1.18]  | 0.18 [0.02; 1.41]  | 0.17 [0.02; 1.37] | SP |

Supplement: Supplementary file 1 [file jcm-14-03396-s001.zip › Figure S4.pdf]

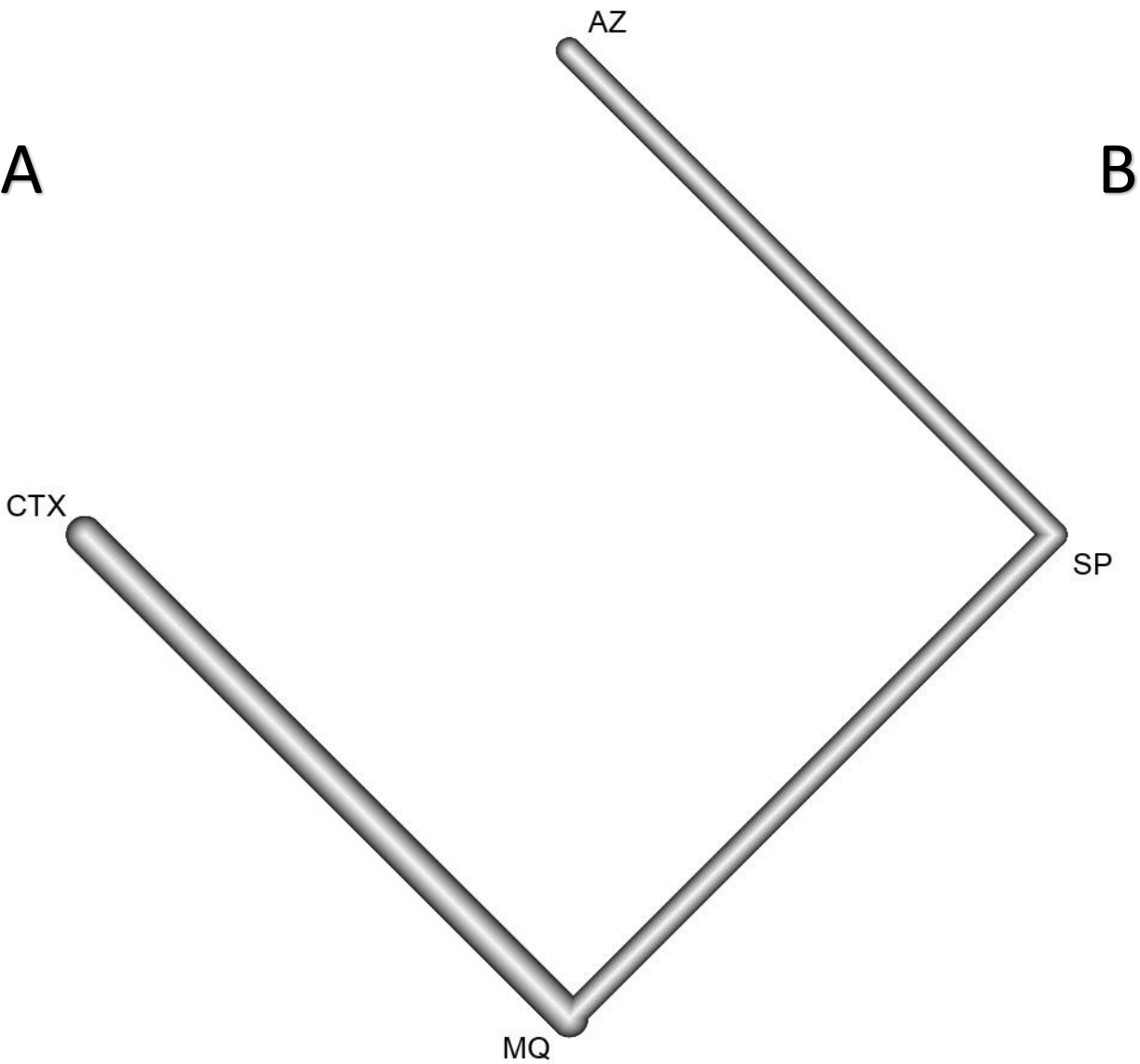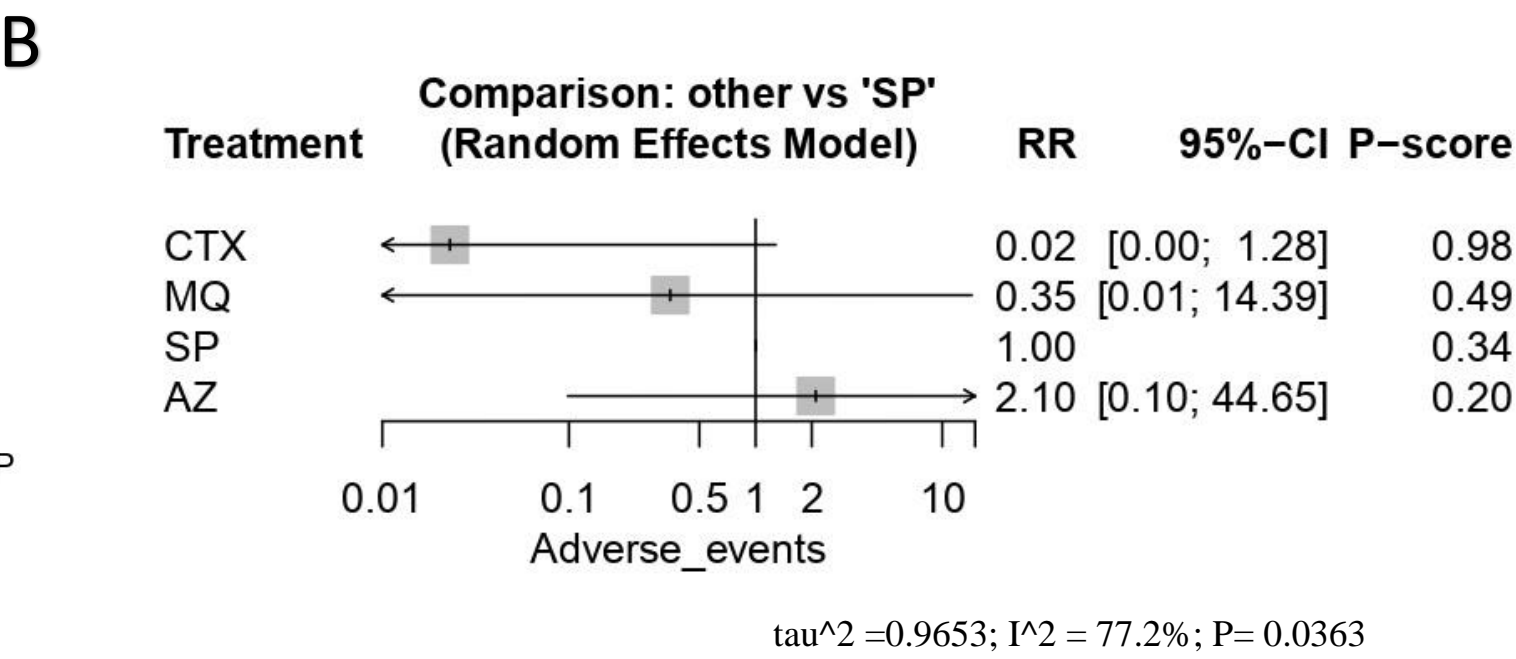

**C**

| CTX               |                    |                    |    |
|-------------------|--------------------|--------------------|----|
| 0.07 [0.01; 0.30] | MQ                 |                    |    |
| 0.02 [0.00; 1.28] | 0.35 [0.01; 14.39] | SP                 |    |
| 0.01 [0.00; 1.71] | 0.17 [0.00; 20.48] | 0.48 [0.02; 10.13] | AZ |

Supplement: Supplementary file 1 [file jcm-14-03396-s001.zip › Figure S5.pdf]

A

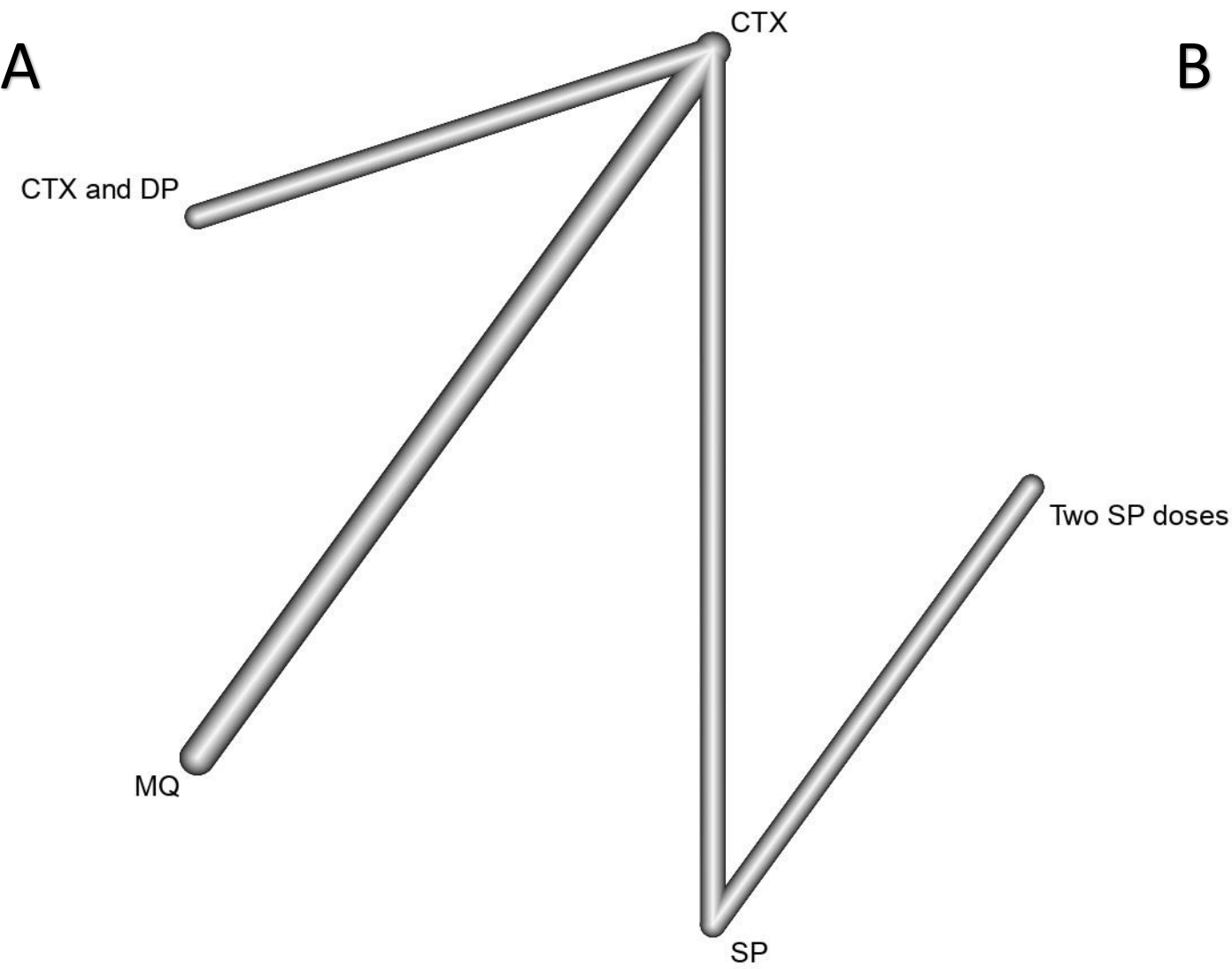

B

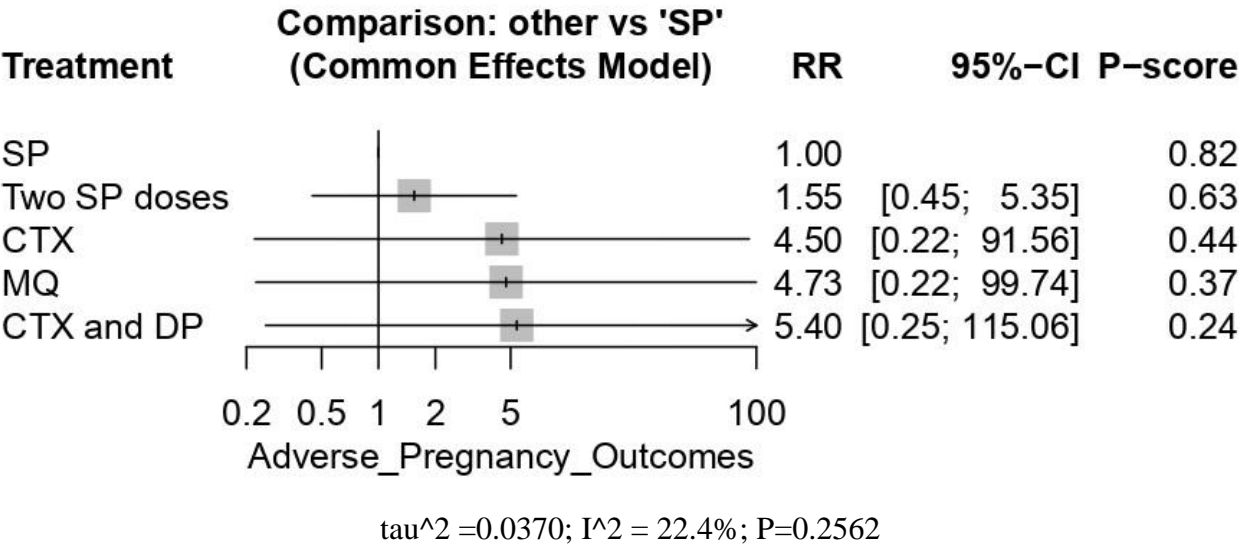

C

| SP                |                   |                   |                   |            |
|-------------------|-------------------|-------------------|-------------------|------------|
| 0.65 [0.19; 2.24] | Two SP doses      |                   |                   |            |
| 0.22 [0.01; 4.53] | 0.34 [0.01; 8.95] | CTX               |                   |            |
| 0.21 [0.01; 4.45] | 0.33 [0.01; 8.78] | 0.95 [0.60; 1.50] | MQ                |            |
| 0.19 [0.01; 3.95] | 0.29 [0.01; 7.78] | 0.83 [0.49; 1.41] | 0.88 [0.44; 1.76] | CTX and DP |

Supplement: Supplementary file 1 [file jcm-14-03396-s001.zip › Figure S6.pdf]

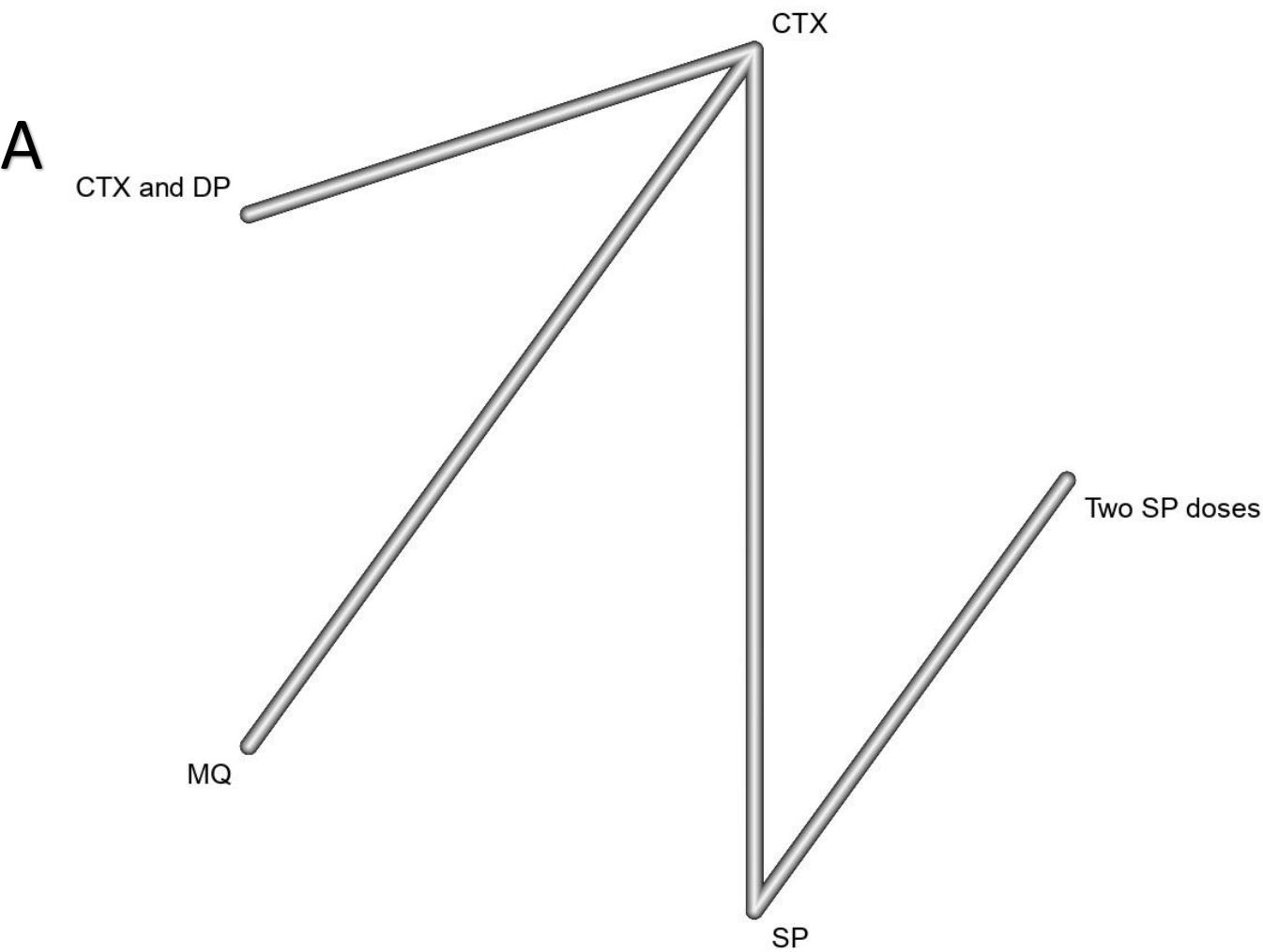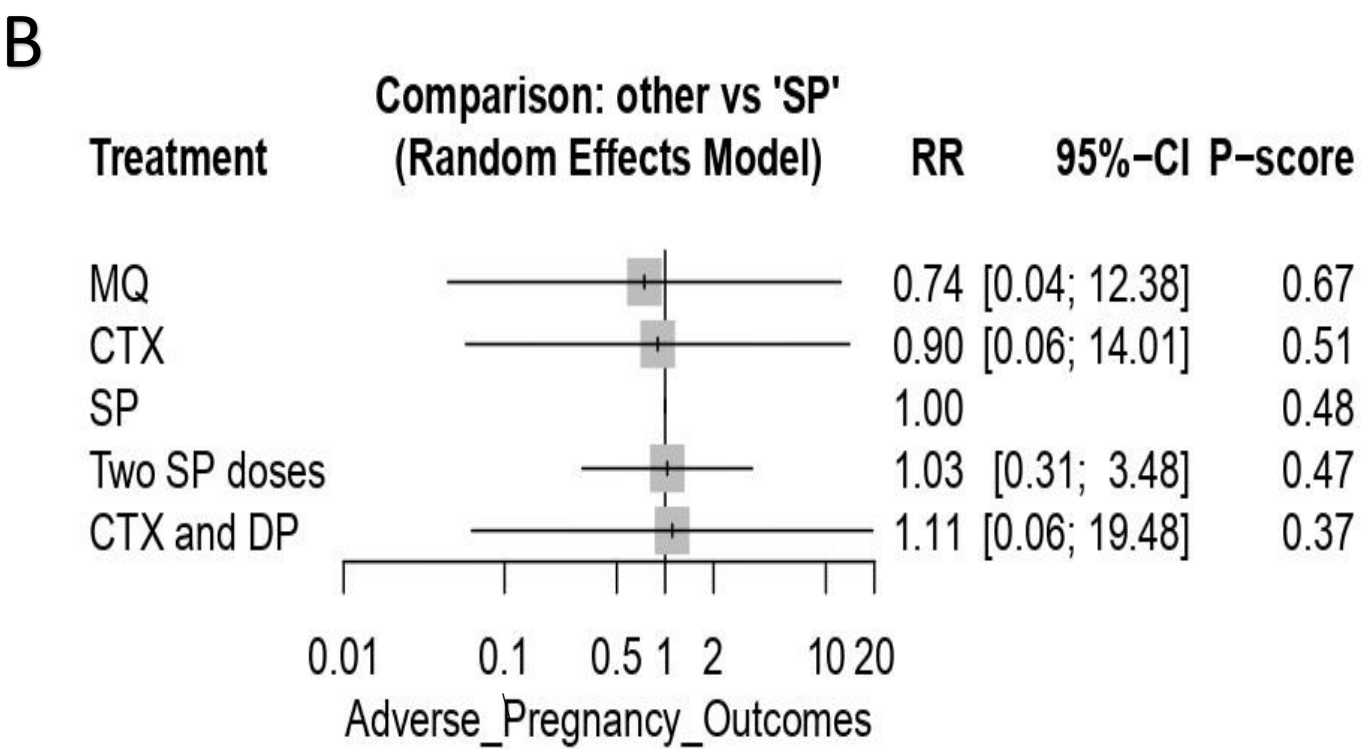

**C**

|                    |                    |                    |                     |                   |
|--------------------|--------------------|--------------------|---------------------|-------------------|
| <b>MQ</b>          |                    |                    |                     |                   |
| 0.83 [0.45; 1.52]  | <b>CTX</b>         |                    |                     |                   |
| 0.74 [0.04; 12.38] | 0.90 [0.06; 14.01] | <b>SP</b>          |                     |                   |
| 0.72 [0.03; 15.45] | 0.87 [0.04; 17.58] | 0.97 [0.29; 3.27]  | <b>Two SP doses</b> |                   |
| 0.67 [0.24; 1.88]  | 0.81 [0.35; 1.86]  | 0.90 [0.05; 15.94] | 0.93 [0.04; 21.03]  | <b>CTX and DP</b> |

Supplement: Supplementary file 1 [file jcm-14-03396-s001.zip › Figure S7.pdf]

A

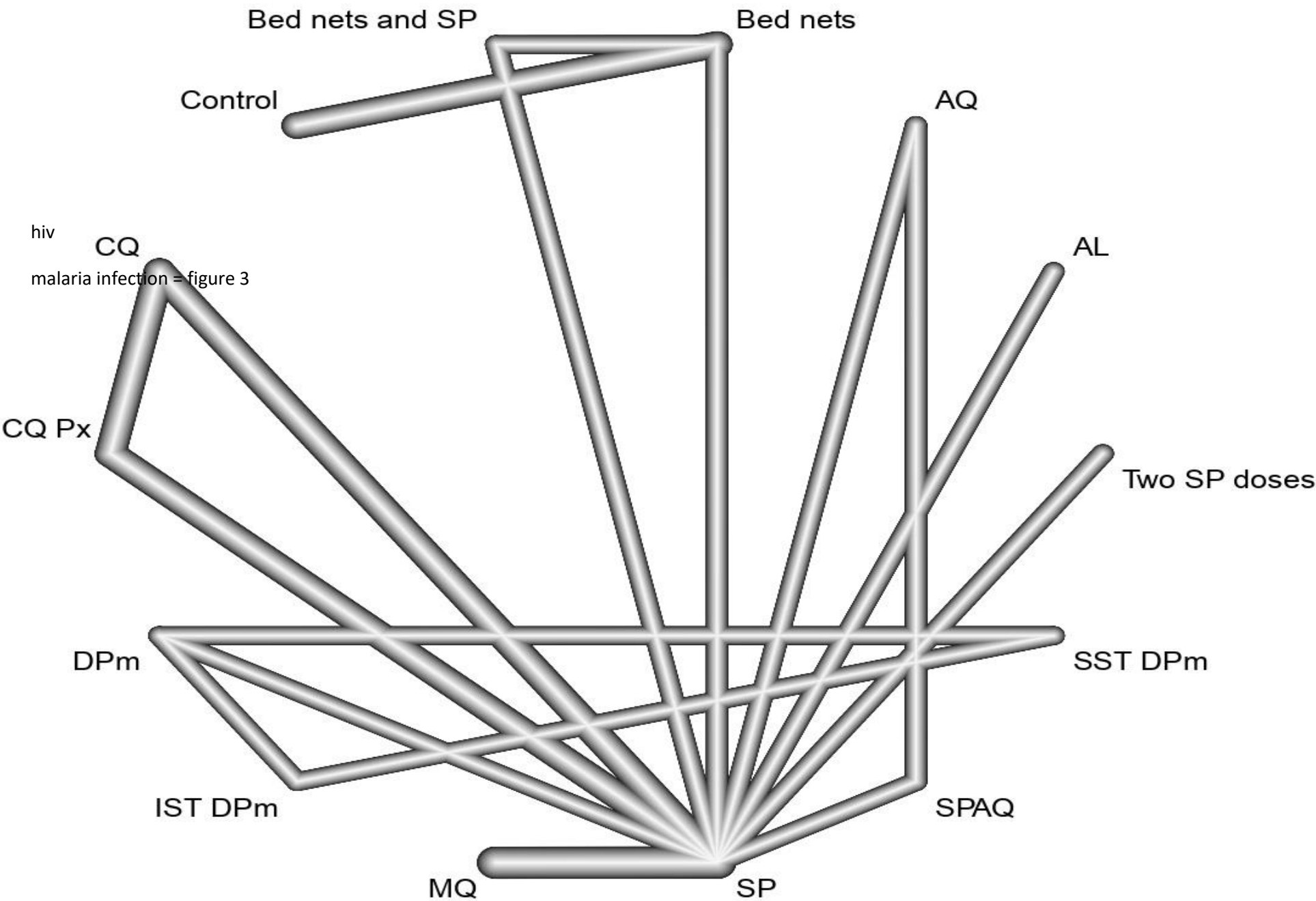

B

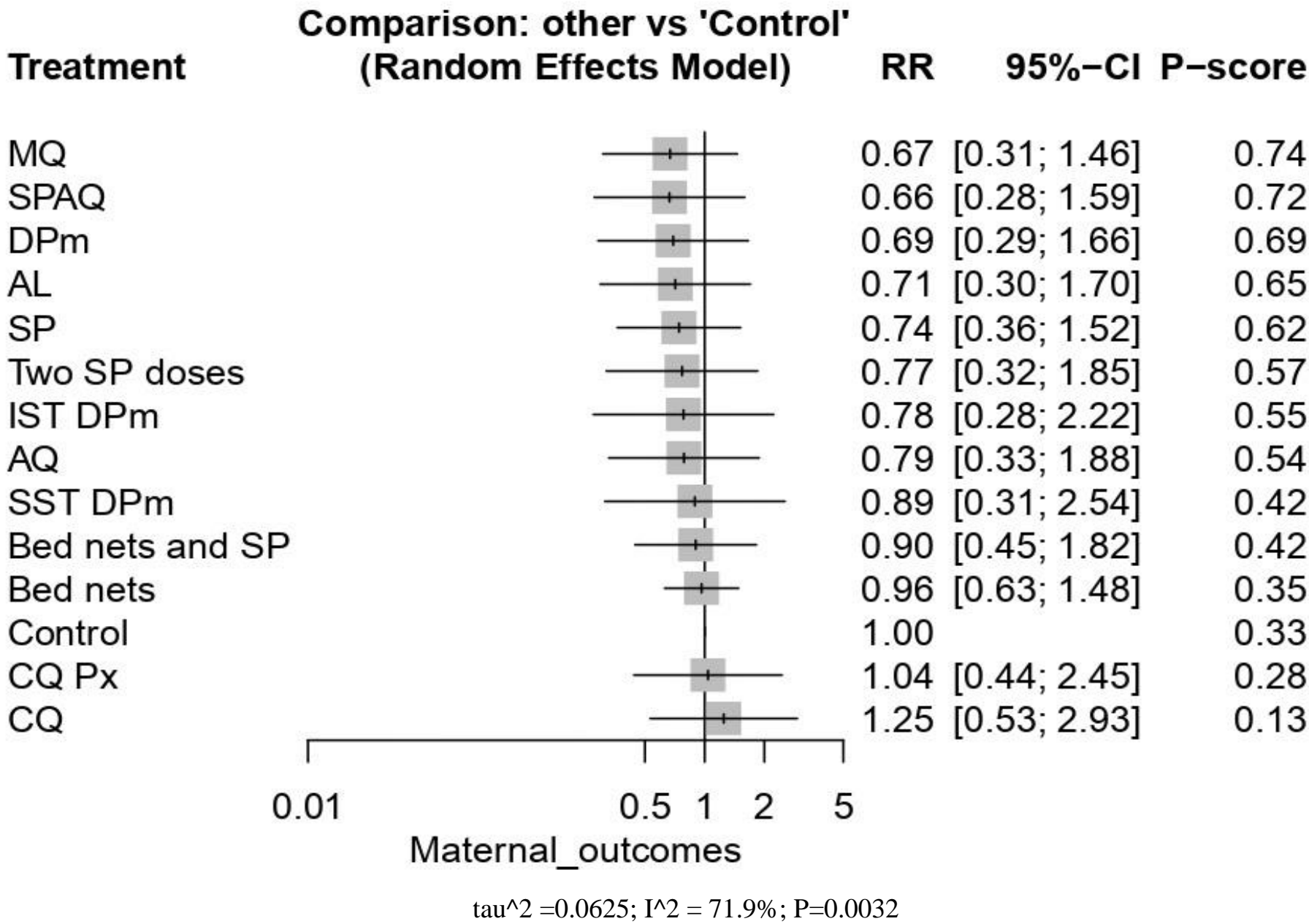

C

|                   |                   |                   |                   |                   |                   |                   |                   |                   |                   |                   |                   |                   |    |
|-------------------|-------------------|-------------------|-------------------|-------------------|-------------------|-------------------|-------------------|-------------------|-------------------|-------------------|-------------------|-------------------|----|
| MQ                |                   |                   |                   |                   |                   |                   |                   |                   |                   |                   |                   |                   |    |
| 1.01 [0.56; 1.81] | SPAQ              |                   |                   |                   |                   |                   |                   |                   |                   |                   |                   |                   |    |
| 0.96 [0.54; 1.73] | 0.96 [0.47; 1.94] | DPm               |                   |                   |                   |                   |                   |                   |                   |                   |                   |                   |    |
| 0.94 [0.52; 1.69] | 0.93 [0.46; 1.89] | 0.98 [0.48; 1.97] | AL                |                   |                   |                   |                   |                   |                   |                   |                   |                   |    |
| 0.90 [0.66; 1.23] | 0.90 [0.54; 1.48] | 0.93 [0.57; 1.54] | 0.96 [0.58; 1.57] | SP                |                   |                   |                   |                   |                   |                   |                   |                   |    |
| 0.87 [0.48; 1.58] | 0.86 [0.42; 1.76] | 0.90 [0.44; 1.84] | 0.92 [0.45; 1.88] | 0.97 [0.58; 1.61] | Two SP doses      |                   |                   |                   |                   |                   |                   |                   |    |
| 0.86 [0.38; 1.95] | 0.85 [0.34; 2.11] | 0.89 [0.50; 1.58] | 0.91 [0.37; 2.26] | 0.95 [0.44; 2.03] | 0.98 [0.39; 2.46] | IST DPm           |                   |                   |                   |                   |                   |                   |    |
| 0.85 [0.47; 1.53] | 0.85 [0.51; 1.39] | 0.88 [0.44; 1.78] | 0.90 [0.45; 1.82] | 0.94 [0.57; 1.55] | 0.98 [0.48; 1.99] | 0.99 [0.40; 2.47] | AQ                |                   |                   |                   |                   |                   |    |
| 0.75 [0.33; 1.70] | 0.74 [0.30; 1.85] | 0.78 [0.44; 1.38] | 0.80 [0.32; 1.97] | 0.83 [0.39; 1.78] | 0.86 [0.34; 2.15] | 0.88 [0.50; 1.53] | 0.88 [0.35; 2.19] | SST DPm           |                   |                   |                   |                   |    |
| 0.74 [0.39; 1.43] | 0.74 [0.34; 1.58] | 0.77 [0.36; 1.65] | 0.79 [0.37; 1.69] | 0.82 [0.46; 1.47] | 0.85 [0.40; 1.84] | 0.87 [0.33; 2.26] | 0.87 [0.41; 1.87] | 0.99 [0.38; 2.57] | Bed nets and SP   |                   |                   |                   |    |
| 0.69 [0.36; 1.33] | 0.69 [0.32; 1.47] | 0.72 [0.34; 1.54] | 0.74 [0.35; 1.57] | 0.77 [0.43; 1.37] | 0.80 [0.37; 1.72] | 0.81 [0.31; 2.10] | 0.82 [0.38; 1.74] | 0.93 [0.36; 2.40] | 0.93 [0.53; 1.63] | Bed nets          |                   |                   |    |
| 0.67 [0.31; 1.46] | 0.66 [0.28; 1.59] | 0.69 [0.29; 1.66] | 0.71 [0.30; 1.70] | 0.74 [0.36; 1.52] | 0.77 [0.32; 1.85] | 0.78 [0.28; 2.22] | 0.79 [0.33; 1.88] | 0.89 [0.31; 2.54] | 0.90 [0.45; 1.82] | 0.96 [0.63; 1.48] | Control           |                   |    |
| 0.64 [0.36; 1.14] | 0.64 [0.32; 1.27] | 0.67 [0.34; 1.33] | 0.68 [0.34; 1.36] | 0.71 [0.44; 1.15] | 0.74 [0.37; 1.48] | 0.75 [0.31; 1.85] | 0.76 [0.38; 1.51] | 0.86 [0.35; 2.11] | 0.87 [0.41; 1.83] | 0.93 [0.44; 1.95] | 0.96 [0.41; 2.27] | CQ Px             |    |
| 0.54 [0.31; 0.94] | 0.53 [0.27; 1.05] | 0.56 [0.28; 1.10] | 0.57 [0.29; 1.12] | 0.59 [0.37; 0.95] | 0.62 [0.31; 1.23] | 0.63 [0.26; 1.53] | 0.63 [0.32; 1.25] | 0.72 [0.29; 1.74] | 0.72 [0.34; 1.51] | 0.77 [0.37; 1.62] | 0.80 [0.34; 1.88] | 0.83 [0.53; 1.31] | CQ |

Supplement: Supplementary file 1 [file jcm-14-03396-s001.zip › Figure S8.pdf]

A

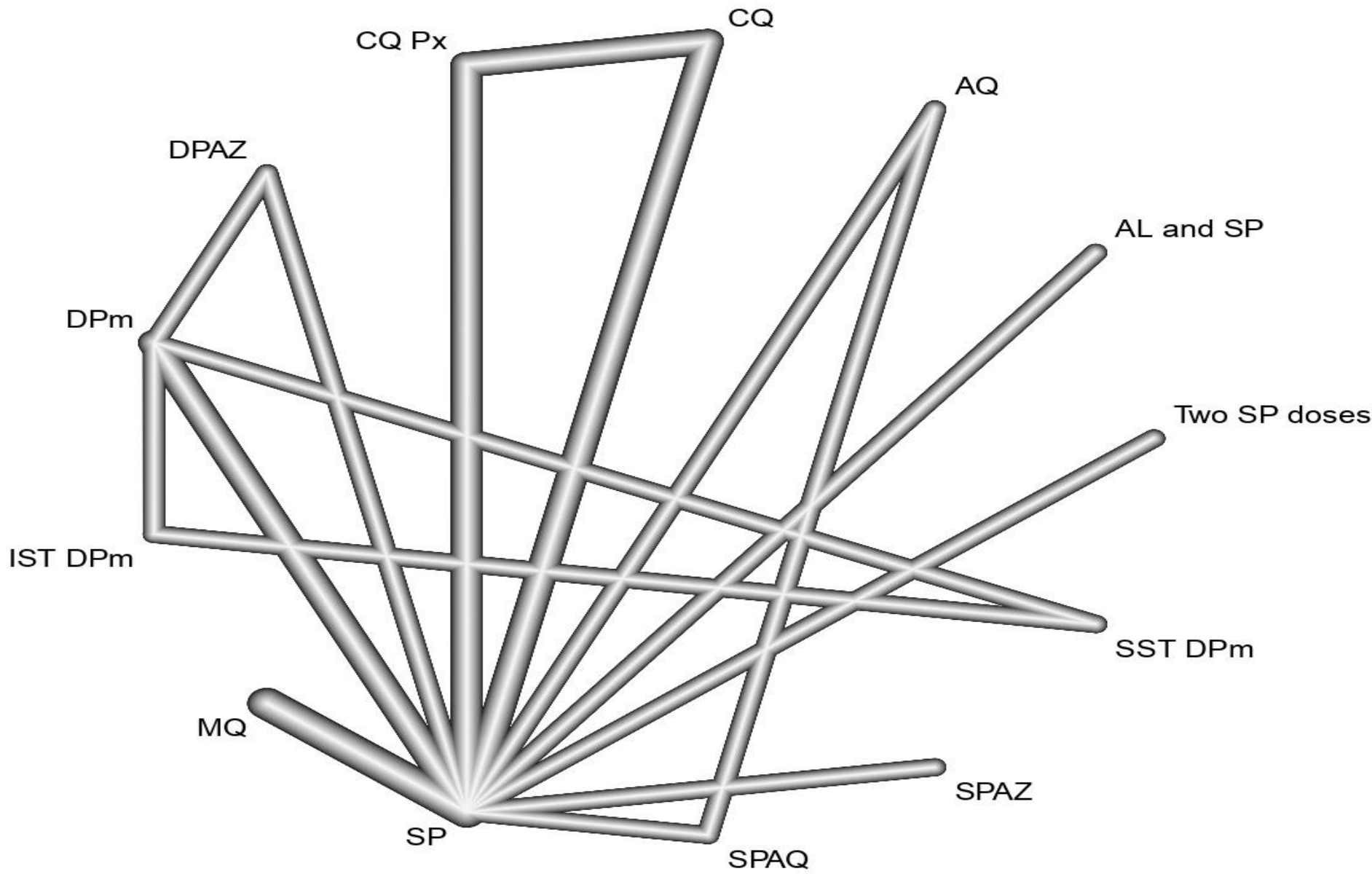

B

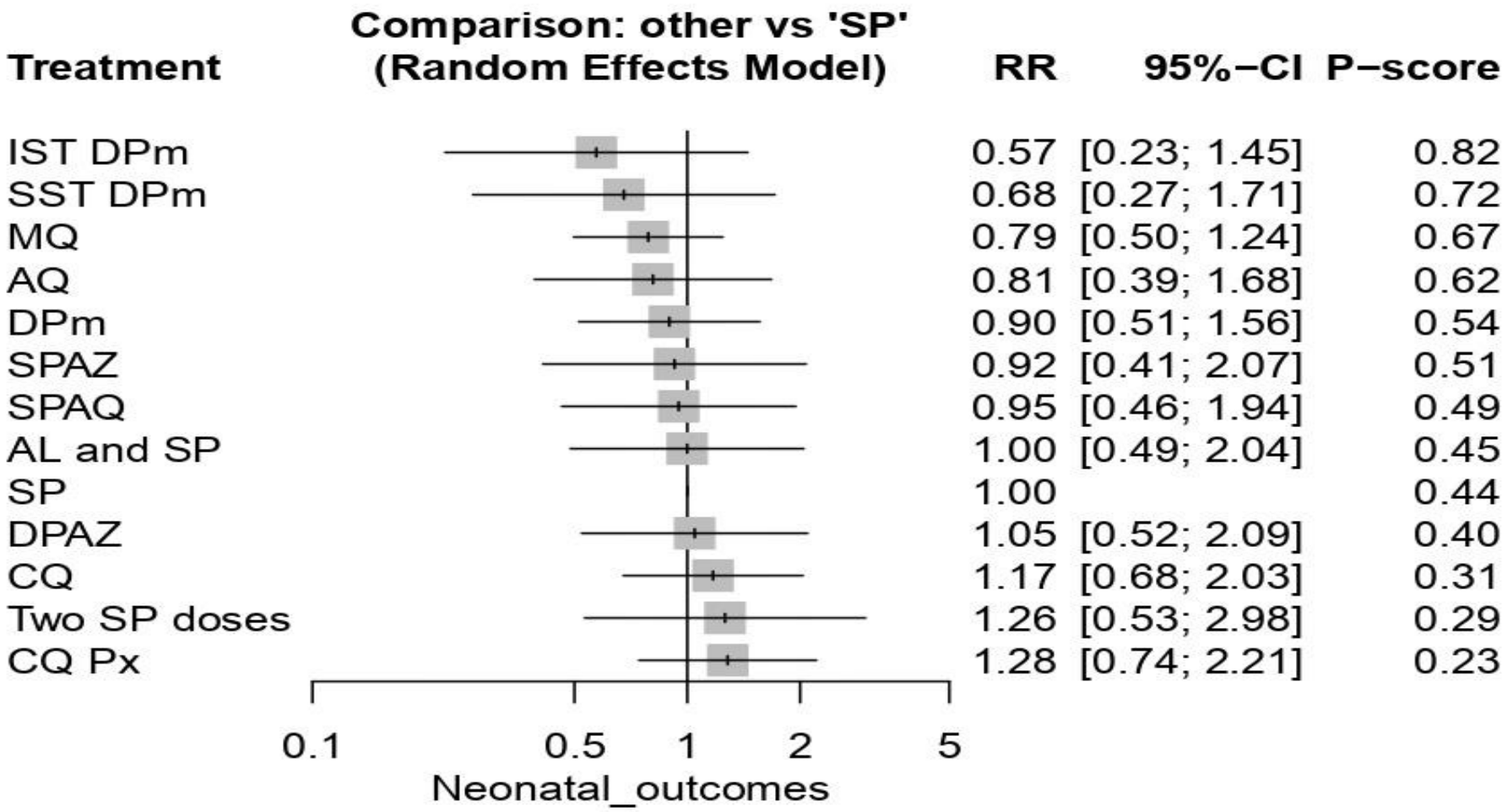

tau^2 =0.1245; I^2 = 79.7%; P=0.0002

C

|                   |                   |                   |                   |                   |                   |                   |                   |                   |                   |                   |                   |       |
|-------------------|-------------------|-------------------|-------------------|-------------------|-------------------|-------------------|-------------------|-------------------|-------------------|-------------------|-------------------|-------|
| IST DPm           |                   |                   |                   |                   |                   |                   |                   |                   |                   |                   |                   |       |
| 0.84 [0.40; 1.78] | SST DPm           |                   |                   |                   |                   |                   |                   |                   |                   |                   |                   |       |
| 0.73 [0.26; 2.05] | 0.86 [0.31; 2.42] | MQ                |                   |                   |                   |                   |                   |                   |                   |                   |                   |       |
| 0.71 [0.22; 2.30] | 0.84 [0.26; 2.71] | 0.97 [0.41; 2.29] | AQ                |                   |                   |                   |                   |                   |                   |                   |                   |       |
| 0.64 [0.30; 1.34] | 0.76 [0.36; 1.59] | 0.88 [0.43; 1.80] | 0.90 [0.36; 2.26] | DPm               |                   |                   |                   |                   |                   |                   |                   |       |
| 0.62 [0.18; 2.12] | 0.73 [0.21; 2.50] | 0.85 [0.34; 2.15] | 0.88 [0.30; 2.60] | 0.97 [0.36; 2.58] | SPAZ              |                   |                   |                   |                   |                   |                   |       |
| 0.60 [0.19; 1.95] | 0.71 [0.22; 2.30] | 0.83 [0.35; 1.94] | 0.85 [0.41; 1.77] | 0.94 [0.38; 2.34] | 0.97 [0.33; 2.87] | SPAQ              |                   |                   |                   |                   |                   |       |
| 0.57 [0.18; 1.85] | 0.68 [0.21; 2.19] | 0.79 [0.34; 1.84] | 0.81 [0.29; 2.25] | 0.90 [0.36; 2.22] | 0.93 [0.31; 2.72] | 0.95 [0.34; 2.62] | AL and SP         |                   |                   |                   |                   |       |
| 0.57 [0.23; 1.45] | 0.68 [0.27; 1.71] | 0.79 [0.50; 1.24] | 0.81 [0.39; 1.68] | 0.90 [0.51; 1.56] | 0.92 [0.41; 2.07] | 0.95 [0.46; 1.94] | 1.00 [0.49; 2.04] | SP                |                   |                   |                   |       |
| 0.55 [0.20; 1.51] | 0.65 [0.24; 1.78] | 0.75 [0.33; 1.72] | 0.77 [0.28; 2.11] | 0.86 [0.43; 1.70] | 0.88 [0.30; 2.56] | 0.91 [0.33; 2.46] | 0.95 [0.35; 2.58] | 0.96 [0.48; 1.91] | DPAZ              |                   |                   |       |
| 0.49 [0.17; 1.44] | 0.58 [0.20; 1.70] | 0.67 [0.33; 1.37] | 0.69 [0.28; 1.72] | 0.76 [0.35; 1.67] | 0.79 [0.30; 2.10] | 0.81 [0.33; 2.00] | 0.85 [0.35; 2.10] | 0.85 [0.49; 1.48] | 0.89 [0.37; 2.16] | CQ                |                   |       |
| 0.45 [0.13; 1.61] | 0.54 [0.15; 1.90] | 0.62 [0.24; 1.66] | 0.64 [0.21; 1.98] | 0.71 [0.25; 1.98] | 0.73 [0.23; 2.39] | 0.75 [0.25; 2.31] | 0.79 [0.26; 2.43] | 0.79 [0.34; 1.88] | 0.83 [0.27; 2.51] | 0.93 [0.33; 2.58] | Two SP doses      |       |
| 0.45 [0.15; 1.31] | 0.53 [0.18; 1.55] | 0.61 [0.30; 1.25] | 0.63 [0.25; 1.57] | 0.70 [0.32; 1.52] | 0.72 [0.27; 1.91] | 0.74 [0.30; 1.82] | 0.78 [0.32; 1.91] | 0.78 [0.45; 1.34] | 0.82 [0.34; 1.97] | 0.91 [0.53; 1.57] | 0.98 [0.36; 2.73] | CQ Px |

Supplement: Supplementary file 1 [file jcm-14-03396-s001.zip › Figure S9.pdf]
